# Genetic basis of phenotypic plasticity and genotype × environment interactions in a multi-parental tomato population

**DOI:** 10.1093/jxb/eraa265

**Published:** 2020-05-31

**Authors:** Isidore Diouf, Laurent Derivot, Shai Koussevitzky, Yolande Carretero, Frédérique Bitton, Laurence Moreau, Mathilde Causse

**Affiliations:** 1 INRAE, GAFL, Monfavet, France; 2 GAUTIER Semences, route d’Avignon, Eyragues, France; 3 Hazera – Seeds of Growth, Berurim M.P Shikmim, Israel; 4 UMR GQE-Le Moulon, INRA, CNRS, AgroParisTech, Université Paris-Saclay, Gif-sur-Yvette, France; 5 CSIRO Agriculture and Food, Australia

**Keywords:** Abiotic stresses, genotype × environment interaction (G×E), MAGIC population, phenotypic plasticity, tomato, QTL

## Abstract

Deciphering the genetic basis of phenotypic plasticity and genotype × environment interactions (G×E) is of primary importance for plant breeding in the context of global climate change. Tomato (*Solanum lycopersicum*) is a widely cultivated crop that can grow in different geographical habitats and that displays a great capacity for expressing phenotypic plasticity. We used a multi-parental advanced generation intercross (MAGIC) tomato population to explore G×E and plasticity for multiple traits measured in a multi-environment trial (MET) comprising optimal cultural conditions together with water deficit, salinity, and heat stress over 12 environments. Substantial G×E was observed for all the traits measured. Different plasticity parameters were estimated by employing Finlay–Wilkinson and factorial regression models and these were used together with genotypic means for quantitative trait loci (QTL) mapping analyses. In addition, mixed linear models were also used to investigate the presence of QTL × environment interactions. The results highlighted a complex genetic architecture of tomato plasticity and G×E. Candidate genes that might be involved in the occurrence of G×E are proposed, paving the way for functional characterization of stress response genes in tomato and for breeding climate-adapted cultivars.

## Teaser

Measurements of agronomic and fruit quality traits in a multi-environment trial using a multi-parental advanced generation intercross population identify QTLs for plasticity and QTL × environment interactions in response to abiotic stresses in tomato.

## Introduction

Plants are sessile organisms that have to cope with environmental fluctuations to ensure species reproduction and persistence in nature. For a given genotype, the expression of different phenotypes according to the growing environment is commonly called phenotypic plasticity (PP) (Bradshaw, 1965). It offers the possibility to plants to adapt to new environments, notably new locations and changes in climatic conditions or seasonal variations. In agriculture, the range of environmental variation for crop cultivation may also include different cultural practices or growing conditions, leading to the expression of PP in agronomic traits and hence to unstable performance. When different genotypes/accessions are examined for PP within a species, inter-individual variations in their responses usually lead to the common phenomenon of genotype × environment interaction (G×E) (El-Soda *et al.*, 2014). Understanding the genetic mechanisms driving PP and G×E in plants is a crucial step for being able to predict yield performance of crop cultivars and to adapt breeding strategies according to the targeted environments.

The genetic basis of PP in plants has been investigated to assess whether it has its own genetic regulation and hence if it could be directly selected. Three main genetic models have been proposed in the literature as underlying plant PP, widely known as the over-dominance, allelic-sensitivity, and gene-regulatory models (Scheiner, 1993; Via *et al.*, 1995). The over-dominance model suggests that PP is negatively correlated to the number of heterozygous loci (Gillespie and Turelli, 1989), with the heterozygous status being favored by the complementarity of alleles. The allelic-sensitivity and gene-regulatory models are assumed to arise from the differential expression of an allele according to the environment and from epistatic interactions between structural and regulatory alleles, respectively. The latter assumes an independent genetic control of the mean phenotype and plasticity of a trait. Using a wide range of environmental conditions, the prevalence of the allelic-sensitivity or gene-regulatory models in explaining the genetic architecture of PP has been explored in different crop species including barley (Lacaze *et al.*, 2009), maize (Gage *et al.*, 2017; Kusmec *et al.*, 2017), soybean (Xavier *et al.*, 2018), and sunflower (Mangin *et al.*, 2017).

Quantification of PP, however, is often an issue when analysing the genetic architecture of plasticity since different parameters for the estimation of PP are available, as reviewed by Valladares *et al.* (2006). At a population level, when multiple genotypes are screened in different environments different approaches can be used to assess plasticity (Laitinen and Nikoloski, 2019). The most common of these is the joint regression model (Finlay and Wilkinson, 1963) that uses the average performance of the set of tested genotypes in each environment as an index on which the individual phenotypes are regressed. This model, commonly known as the Finlay–Wilkinson regression model, allows the estimation of linear (slopes) and non-linear plasticity parameters (from the residual errors), which presumably have a different genetic basis (Kusmec *et al.*, 2017). If detailed descriptions of the environments are available, the environmental index used in the Finlay–Wilkinson regression model can be replaced by environmental covariates such as stress indexes through factorial regression models (Malosetti *et al.*, 2013). Thus, plasticity can be estimated as the degree of sensitivity to a given stress continuum (Mangin *et al.*, 2017).

Climate change is predicted to increase the frequency and intensity of abiotic stresses with a resulting high and negative impact on crop yield (Zhao *et al.*, 2017). Plants respond to abiotic stresses by altering their morphology and physiology, reallocating energy for growth to defense against stress (Munns and Gilliham, 2015), with consequences on agronomic performance that are apparent and detrimental to productivity. The most common abiotic stresses that have been studied across species are water deficit (WD), salinity stress (SS), and high-temperature stress (HT). The negative impact of these stresses on yield have been highlighted for major cultivated crops; however, positive effects of WD and SS on fruit quality have been observed in fruit trees and some vegetables, notably in tomato (Mitchell *et al.*, 1991; Costa *et al.*, 2007; Ripoll *et al.*, 2014).

Tomato is an economically important crop and a model plant species, which has led to numerous studies that have contributed much to understanding the genetic architecture of the crop and its response to environmental variation. However, most of the studies that have addressed the response of the genetic architecture of tomato to the environment have been conducted on experimental populations exposed to two conditions (i.e. control versus stress). For example, Albert *et al.* (2018) identified different quantitative trait loci (QTL) for the WD response in a bi-parental population derived from a cross of large and cherry tomato accessions. Tomato heat-response QTLs have also been identified in different experimental populations, including both inter- and intraspecific (Grilli *et al.*, 2007; Xu *et al.*, 2017*a*; Driedonks *et al.*, 2018). These studies mostly investigated heat-response QTLs using reproductive traits screened under heat-stress conditions. Villalta *et al.* (2007) and Diouf *et al.* (2018) investigated the response of the genetic architecture of tomato to SS and identified different QTLs for physiological and agronomic traits that were involved in salinity tolerance. However, no QTL study has yet been conducted on tomato plasticity under a multiple-stress design, despite the fact that the coincidence of different stresses is a more realistic scenario in crop cultivation, especially under climate change.

Tomato benefits from the existence of a large panel of genetic resources that have been used in multiple genetic mapping analyses (Grandillo *et al.*, 2013). Bi-parental populations were first used in QTL mapping and permitted the characterization of many QTL that are related to yield, disease resistance, and fruit quality. In the genomic era, new experimental populations have been developed, offering greater power and advantages for the detection of QTLs. These include mutant collections, backcross inbred line (BIL) populations, and multi-parent advanced generation intercross (MAGIC) populations, as described in Rothan *et al.* (2019). The first tomato MAGIC population was developed at INRA-Avignon in France and is composed of about 400 lines derived from an 8-way cross (Pascual *et al.*, 2015). This population shows a wide intraspecific genetic variation under control and stress environments and is highly suitable for mapping QTLs (Diouf *et al.*, 2018).

In the present study, we used this 8-way tomato MAGIC population to evaluate its response in a multi-environment trial (MET). The population was grown in 12 environments that included control conditions and several stresses (WD, SS, and HT), and agronomic traits related to yield, fruit quality, plant growth, and phenology were measured. Different plasticity parameters were computed and used together with mean phenotypes to decipher the genetic control of the response to environmental variation. In addition, multi-environment QTL analysis was performed to detect QTL × environment interactions (QEIs) together with QTL mapping for plasticity traits.

## Materials and methods

### Plant material and phenotyping

The MAGIC population was derived from a cross between eight parental lines that belong to the *Solanum lycopersicum* and *S. lycopersicum* var. *cerasiforme* groups. More details about the population development can be found in Pascual *et al.* (2015). Briefly, the population was composed of about 400 8-way MAGIC lines that underwent three generations of selfing before greenhouse evaluations were carried out. In the current study, a subset of 241–397 lines was grown in each environment (Supplementary Table S1 at *JXB* online).

The full genome of each parental line was resequenced and comparisons with the reference tomato genome (‘Heinz 1706’) yielded 4 million SNPs (Causse *et al.*, 2013). From these polymorphisms, a genetic map of 1345 discriminant SNPs was developed (Pascual *et al.*, 2015) and used in the present study for the QTL analysis.

### Experimental design

The MAGIC population was grown in three different geographical regions (France, Israel, and Morocco) and four specific stress treatments were applied. In a given trial, any stress treatment was applied alongside a control trial (Supplementary Table S1). Treatments consisted of water deficit (WD), two levels of salinity (low and high salinity; LS and HS, respectively), and high-temperature (HT) stress. Water deficit was applied by reducing the irrigation by ~70% and ~30% according to the reference evapotranspiration in Israel in 2014 and 2015, respectively, and by 50% in Morocco in 2015. The salinity treatment was managed as described in Diouf *et al.* (2018) and the mean electrical conductivity (Ec) of the substrate in Morocco in 2016 was 3.76 and 6.50 dS m^–1^ for LS and HS, respectively, while the Ec under control conditions in Morocco 2015 was ~1.79 dS m^–1^. For the HT stress, plants were sown during the late spring and phenotyped in the summer of 2014 in Israel (environment HIs14; Supplementary Table 1) and the summer of 2017 in France (HAvi17). During the HT treatments, the greenhouse vent opening was managed throughout the entire growing season, with vents only opened when temperatures rose above 25 °C. The average mean/maximum temperatures determined on the basis of daily measurements were 26/34 °C for HAvi17 and 33/48 °C for HIs14. Apart from the stress treatments, local conventional cultural conditions were applied in all cases, as described in Diouf *et al.* (2018).

‘Environments’ were considered as any combination of a geographical region, the year of the trial, and an applied treatment (Supplementary Table S1). Climatic sensors were installed in the greenhouses and climatic parameters were recorded hourly in all the environments. From the climatic parameters, seven environmental covariates were defined (Supplementary Fig. S1), namely the temperature parameters of mean, minimal, and maximal daily temperatures, and thermal amplitude, the sum of degree-days (SDD), the vapour-pressure deficit (Vpd, in kPa), and the relative humidity (RH) within the greenhouse. To characterize the environments, every covariate was calculated during the period covering the flowering time of the population on the fourth truss. Indeed, the phenotypic data analysed here were mostly recorded on the fourth and fifth trusses (Supplementary Table S2) and high correlations were found between the covariates calculated for each period. However, it should be noted that for traits that are determined by early developmental stages, the covariates calculated based on the flowering-time window might underestimate the amount of G×E. Hierarchical clustering was performed with the ‘FactoMineR’ R package (Lê *et al.*, 2008) using the environmental parameters to group environments according to their similarity regarding the within-greenhouse climatic conditions.

The MAGIC population, the eight parental lines, and the four first-generation hybrids (one hybrid per 2-way cross) were evaluated for fresh fruit weight (FW) by determining the mean value of the fruits from the third and/or fourth plant truss in each environment. Phenotypic data were recorded across the different environments for nine supplementary traits that were related to (1) fruit quality: fruit firmness (firm) and soluble solid content (SSC); (2) plant phenology: flowering time (flw), number of flowers (nflw), and fruit setting (fset); and (3) plant development: stem diameter (diam), leaf length (leaf), plant height (height), and fruit number (nfr). Details about the phenotyping measurements are given in Supplementary Table S2. At least two plants per MAGIC line were replicated in each environment, except for Avi17 (control conditions) where the phenotype was recorded from measurements on a single plant. Parents and hybrids had more replicates per genotype (at least two) and served as control lines to measure within-environment heterogeneity.

### Evaluation of G×E and heritability

Data were first analysed separately in each environment to remove outliers and to correct for spatial heterogeneity within the environment. Equation (1), below, was applied to test for micro-environmental variation within the greenhouse, where *y*_*ijk*_ represents the phenotype of the individual *i*, located in row *j* and position *k* in the greenhouse, μ is the overall mean, and $C_{i}$ and $L_{i}$ represent the fixed effect of the control lines and the random effect of the MAGIC lines, respectively. In this model, $t_{i}$ is an index of 0 or 1, defined to distinguish between the MAGIC and control lines, and $\varepsilon_{ijk}$ is the random residual error.

$$\begin{array}{l} y_{ijjk}=\;\mu +\;C_{i}.t_{i}+\;L_{i}.\left(1-t_{i}\right)+\;R_{j}+P_{k}+\;\varepsilon_{ijk}\; \end{array}$$ (1)

For every trait where row ($R_{j}$) and/or position ($P_{k}$) effects were significant, required corrections were applied by removing the best linear unbiased prediction (BLUP) of the significant effects from the raw data. The corrected data were gathered and used in eqn (2) in order to estimate the broad-sense heritability ($H^{2}$) and the proportion of variance associated with G×E ($prop.{\sigma^{2}}_{GxE}$).

$$\begin{array}{l} y_{ij}=\;\mu +E_{j}+C_{i}.t_{i}+C\times E_{ij}.t_{i}+\;L_{i}.\left(1-t_{i}\right)+\;L\times E_{ij}.\left(1-t_{i}\right)+\;\varepsilon_{ij} \end{array}$$ (2)

Where $y_{ij}\;$ represents the phenotype of the individual *i* in environment *j*, and *C*×*E*_*ij*_ and *L*×*E*_*ij*_ are the fixed control lines × environment interaction effect and the random MAGIC lines × environment interaction effect, respectively. Within a given environment, random residuals error terms were assumed to be independent and distributed identically with a variance specific to each environment. The proportion of the total genotypic and G×E variance explained by the model was then calculated as: $prop.{\sigma^{2}}_{G\times E}=\;{\sigma^{2}}_{L\times E}/({\sigma^{2}}_{L}+{\sigma^{2}}_{L\times E})$. The significance of G×E was tested with a likelihood ratio test (at the 5% level) between the models with and without G×E. The broad-sense heritability at the whole design level (*H*^2^) was derived from the variance components of eqn (2) and calculated as:

$$\begin{array}{l} H^{2}=\;\;\;{\sigma^{2}}_{L}/({\sigma^{2}}_{L}+\frac{{\sigma^{2}}_{L\times E}}{nb.E}+\;\;\;\frac{{\sigma^{2}}_{E}}{nb.R}) \end{array}$$

where ${\sigma^{2}}_{L}$and ${\sigma^{2}}_{LxE}$are the variance components associated with the MAGIC lines and the MAGIC lines × environment interaction effects, respectively, *nb.E* represents the number of environments (e.g. 12 for FW) and *nb.R* represents the mean number of replicates over the whole design, and ${\sigma^{2}}_{E}\;$ is the mean environmental variance, i.e. $\overset{}{\sum }{\sigma^{2}}_{Ej}/nb.E$

### Phenotypic plasticity

Three different parameters of plasticity were estimated using the Finlay–Wilkinson regression model and a factorial regression model, which were performed using the ordinary least-squares method (OLS).

In the Finlay–Wilkinson model (eqn 3), $y_{ij}$ is the phenotype (mean values per environment and genotype) and μ is the general intercept. $G_{i}$ and $E_{j}$ are the effects of the MAGIC line *i* and environment *j*, respectively, and $\left(1+\beta_{i}\right)$ represents the regression coefficient of the model, which measures genotypic sensitivity to the environment for each line and represents a combination of the population mean response and the genotype-specific response.

$$\begin{array}{l} y_{ij}=\;\mu +G_{i}+(1+\beta_{i})\times E_{j}+\varepsilon_{ij} \end{array}$$ (3)

Environments are described here as an index that represents the ‘quality’ of the environment (i.e. the average performance of all genotypes in a given environment). $\varepsilon_{ij}$ is an error term including the G×E and $\varepsilon_{ij}\;$~ N (0, σ ^2^R). Three parameters were estimated from eqn (3): (i) the genotypic mean, which is equivalent to $\mu +G_{i}$ and represents the average performance of a genotype considering all environments; (ii) the 1+β _i_ term (slope), which corresponds to the genotypic response to the environments; and (iii) the variance (VAR) of the $\varepsilon_{ij}$ term, which is a measurement of non-linear plasticity (Kusmec *et al.*, 2017). All these parameters were then used to characterize the genotypes according to their individual performance and their stability in the MAGIC-MET design. For every trait, reaction norms were then computed from eqn (3).

The factorial regression model (eqn 4) was further applied to describe G×E through the genotypic response to the different environmental covariates (Tmin°, Tmax°, Tm°, Amp.Th°, Vpd, RH, and SDD). The environmental covariates defined from the daily recorded climatic variables in the greenhouses were used for this purpose. For each trait, the most significant environmental covariate (*P*-value significant at *α*=5%) was first identified (by successively testing the significance of each single covariate) and then used as an explanatory variable, represented by $Cv_{j}$ in the equation.

$$\begin{array}{l} y_{ij}=\;\mu +G_{i}+E_{j}+\alpha_{i}\times Cv_{j}+\varepsilon_{ij} \end{array}$$ (4)

The $\alpha_{i}$ term was extracted and considered as a third plasticity parameter (SCv), representing the genotypic sensitivity to the most impacting environmental covariate for each trait. This measurement of plasticity is of interest as it allows the identification of the direction and the intensity of each MAGIC line’s sensitivity to a meaningful environmental covariate. Throughout the rest of this article, the ‘slope’ and ‘VAR’ estimated from the Finlay–Wilkinson model and ‘SCv’ from the factorial regression model will be considered as plasticity phenotypes, all of these parameters being trait-specific. The genotype $G_{i}$ and environment $E_{j}$ effects in eqns (3) and (4) are considered as fixed effects.

### Linkage mapping on the genotypic mean and plasticity phenotypes

Linkage mapping was carried out with a set of 1345 SNP markers selected from the genome resequencing of the eight parental lines. All the MAGIC lines were genotyped for these SNPs, and at each position the founder haplotype probability was predicted with the function *calc_genoprob* in the R/qtl2 package (Broman *et al.*, 2019). The founder probabilities were then used with the Haley–Knott regression model implemented in R/qtl2 for detection of QTLs. The response variables were the genotypic means, slope, VAR, and SCv for each trait. To test for significance, the threshold for all phenotypes was set to a LOD threshold of –log_10_(α/number of SNPs), where α was fixed at the 5% level. The VAR plasticity parameter was log-transformed for all traits except fset (square-root transformation) to meet normality assumptions before the QTL analysis. The function *find_peaks ()* of the R/qtl2 package was used to detect all peaks exceeding the defined threshold, and the LOD score was dropped by two and one units to separate two significant peaks as distinct QTLs and to define the confidence interval (CI) of the QTLs, respectively.

### QTL × environment interaction (QEI) analysis

The strength of QTL dependence on the environment was tested by identifying QTLs that significantly interacted with the environment (QEIs). Two multi-environment forward–backward models (eqns 5 and 6) were used to test the effect of the marker × environment interaction at each marker position:

$$\begin{array}{l} y_{ij}=\;\mu +E_{j}+\underset{p=1}{\overset{8}{\sum }}\;\alpha_{kp}*x_{ikp}\;+\underset{p=1}{\overset{8}{\sum }}\;\beta_{kpj}.\;x_{ikp}+G_{i}+\epsilon_{ij}\;\;\; \end{array}$$ (5)

$$\begin{array}{l} y_{ij}=\;\mu +E_{j}+\underset{p=1}{\overset{8}{\sum }}\;\beta_{kpj}.\;x_{ikp}+G_{i}+\epsilon_{ij} \end{array}$$ (6)

$y_{ij}$ represents the phenotype (mean value per genotype and per environment), $E_{j}$ reflects the fixed environment effect, $\alpha_{kp}$ and β _*kpj*_ represent the main and interactive parental allelic effects (*p*), respectively, at marker *k* and in environment *j* for β _*kpj*_, $x_{ikp}$ is the probability of the parental allele’s origin for the MAGIC line *i*, $G_{i}$ represents a random genotype effect and the residual errors (ε _*ij*_) including a part of the G×E that is not explained by the detected QTLs are specific to each environment, ε _*ij*_ ~ N (0, *σ*^*2*^*Rj*).

Significant QEI were identified in a two-step procedure. First, the main QTL and the QEI effects were tested separately using eqn (5). The QTL detection process was adapted from the script proposed by Giraud *et al.* (2017). Every marker showing a significant main QTL or QEI was added as a fixed cofactor and the significance of the remaining markers was tested again until no more significant markers were found. All markers selected as cofactors were then jointly tested in the backward procedure, and only significant QEIs after the backward selection are reported. The second procedure used eqn (6) to identify QEIs and consisted of a slight modification of eqn (5) where this time β _*kpj*_ represents the global (main + interactive) effect of the marker. This allowed the identification of markers that had a main QTL effect or a QEI just below the detection threshold but whose global effect was significant when the two components were tested jointly. To determine the threshold level for QEI detection, permutation tests were performed 1000 times on the adjusted means with the function *sim.sightr* of the mpMap 2.0 R package (Huang and George, 2011).

### Data availability

The phenotypic data, average climatic parameters, and genotypic information described in the present study are available at https://doi.org/10.15454/UVZTAV. The custom scripts used for the two-stage analysis and QEI modelling are also provided.

## Results

### Environment description

**Fig. 1.**
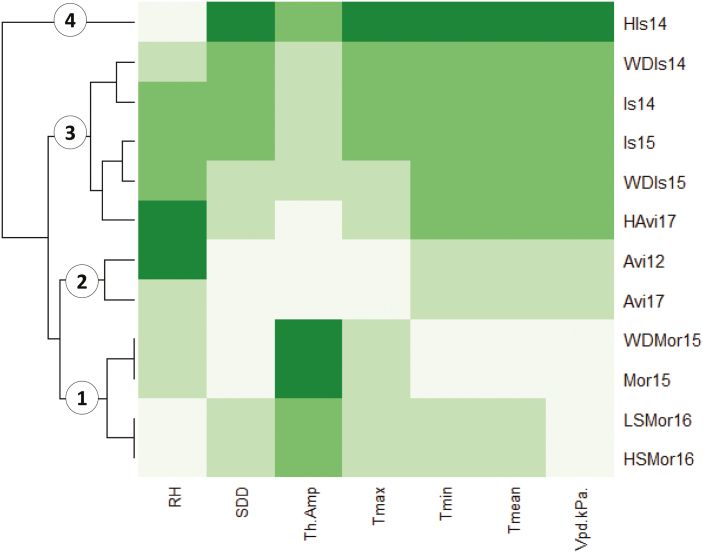
Clustering of the experimental environments according to the seven environmental covariates that were measured during the vegetative and flowering stages. RH, relative humidity; SDD, sum of degree-days; ThAmp, thermal amplitude; Tmax, maximum daily temperature; Tmin, minimum daily temperature; Tmean, mean daily temperature; Vpd.KPa, vapour-pressure deficit. The identifiers for the different environments are listed in Supplementary Table S1.

The 12 environmental conditions were described by the daily climatic parameters that were recorded until the end of flowering of the fourth truss. Seven environmental covariates were selected, according to which the environments clustered into four groups (Fig. 1). The first included all the trials from Morocco that were characterized by high thermal amplitude and low Vpd. The control environments in France (Avi12 and Avi17) clustered together in the 2nd group, and were defined by low maximal temperatures and high relative humidity. HIs14 clustered alone in the 4th group and formed the most extreme environment, showing very high temperatures and a dry climate with low relative humidity. The remaining environments clustered together in the 3rd and most disparate group.

Phenotypic distributions were plotted for each trait for the environment in which they were evaluated (Supplementary Fig. S2) and for some traits (firm, height, nflw, and leaf) they showed a distribution that was in accordance with the clustering of the environments. Other traits such as FW, nfr, SSC, and fset showed a distribution pattern with relatively high within-group variability, notably for environments clustering in group 1 from Morocco.

### G×E in the MAGIC population

Genotype × environment interaction analysis was carried out after correcting the data for micro-environmental heterogeneity and removing outliers. As a first step, variance analysis was conducted using the ASReml-R package, and the variance components from eqn (2) were used to estimate the proportion of G×E variance ($prop.{\sigma^{2}}_{GxE}$) and heritability at the whole-design level ($H^{2}$). Significant G×E was found for every trait and $prop.{\sigma^{2}}_{GxE}$ varied from 0.15 (nflw) to 0.68 (leaf). Although G×E was significant, 7 out of the 10 measured traits showed a higher proportion of genotypic variance compared to G×E (Supplementary Table S3). $H^{2}$ was largely variable according to the trait, varying from 0.18 (nfr) to 0.77 (flw). Its calculation took into account the residual environment-specific variance, which showed different ranges according to the trait, lowering the heritability of traits such as nfr and fset (Supplementary Table S3). Furthermore, $H^{2}$ at the whole-design level was lower than the heritability computed in single environment (Supplementary Fig. S3).

The proportion of G×E that could be predicted by the environmental covariates was then assessed following the factorial regression model (eqn 4). Different environmental covariates significantly explained the G×E across traits (Supplementary Fig. S4). Considering only the most significant covariate, between 18% (FW) to 47% (fset) of the G×E (proportion of the sum of squares) could be reliably attributed to the responses of the genotypes to the climatic parameters measured within the greenhouses. To perform the factorial regression model (eqn 4), the most important environmental covariate was first identified for each trait (Supplementary Fig. S4). For example, the growth traits height and leaf were mostly affected by the thermal amplitude and maximal temperature, respectively, while the yield component traits FW and nfr were particularly sensitive to the sum of degree-days. The vapour pressure deficit (Vpd) was the most important environmental factor affecting firm, fset, and SSC. Flowering time (flw) and nflw were mostly affected by the minimal temperature and relative humidity, respectively. Stem diameter was the only trait for which none of the environmental covariates significantly affected the trait. However, the limitation of using a single covariate at a time in factorial regression models is that G×E is only described according to one environmental factor. Mangin *et al.* (2017), for example, used three environmental covariates in a multi-stress experimental design to characterize G×E in sunflower, which could represent a more realistic scenario of what would be expected in the field. The factorial regression model in our study therefore did not address the limitation of single-stress studies.

### Phenotypic plasticity

**Fig. 2.**
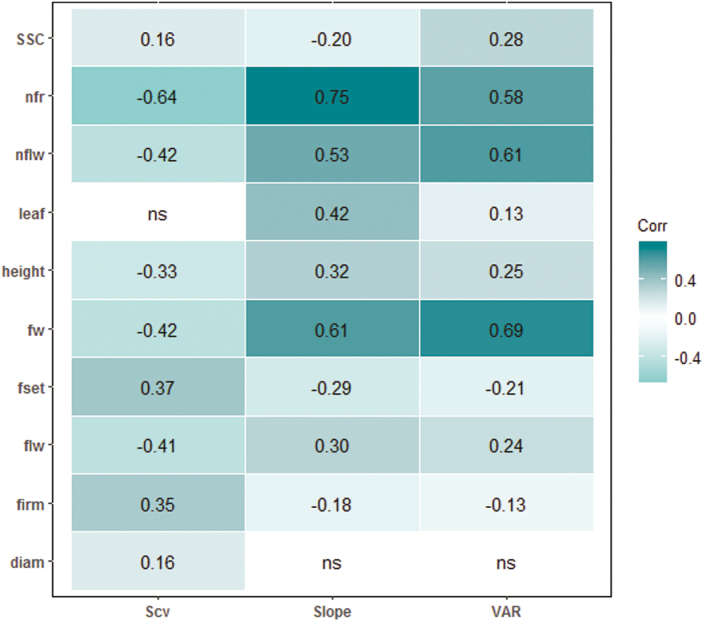
Pearson’s correlations between the genotypic means of traits and plasticity parameters across the different environments. The traits are listed in Supplementary Table S2. For the plasticity parameters, ‘SCv’ represents the genotypic sensitivity of the trait to the most impacting environmental covariate (eqn 4); ‘Slope’ represents the genotypic response to the different environments (slope of eqn 3); and ‘VAR’ is the variance (eqn 3).

Three different parameters were used to quantify phenotypic plasticity in our MAGIC-MET design. For each trait, the slope and VAR from the Finlay–Wilkinson regression model and the genotypic sensitivity to the most important environmental covariate (SCv) from the factorial regression model were extracted. A large genetic variability was observed for the plasticity of all traits (Supplementary Fig. S5). In addition, significant correlations were found between the mean phenotypes and plasticity parameters for most of the traits (Fig. 2). The best mean-performing genotypes were usually the most responsive to environmental variation, as highlighted by the positive correlation between the genotypic means and the slope from the Finlay–Wilkinson regression model.

### QTL mapping

**Fig. 3.**
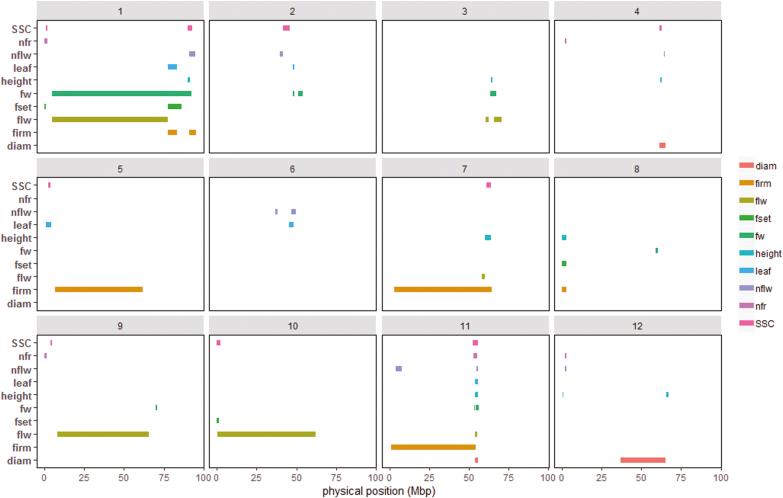
Representation of plasticity QTLs within the genome. The chromosome numbers are indicated above each diagram and the traits are listed in Supplementary Table S2.

We used the genotypic means and plasticity measurements for every trait as input phenotypes to decipher the genetic architecture of the response of tomato to abiotic stresses. Considering the 10 traits evaluated, a total of 104 unique QTLs were identified for the genotypic means and the plasticity parameters (Supplementary Table S4). The proportion of QTLs shared between the two was ~21%, which was lower than the QTLs that were specific for plasticity or the mean (79%). Considering only the 63 plasticity QTLs, 11 and seven QTLs were detected only with the SCv and VAR plasticity parameters, respectively, while the others were either detected with by the slope or by a combination of the different plasticity parameters. Plasticity QTLs were detected on every chromosome (Fig. 3); however, chromosome 1 showed the highest number with 12 plasticity QTLs. In this chromosome, plasticity QTLs were detected at least once for every trait. By performing a Chi-square test considering all the 63 plasticity QTLs, we observed that the presence of plasticity QTLs was higher than would be expected by chance on chromosomes 1 and 11. chromosome 11 carried a total of 11 plasticity QTLs and, interestingly, all these (except ppnflw11.1) co-localized in a short region of the chromosome between 52–55 Mbp. Chromosomes 5, 6, and 10 showed the lowest number of plasticity QTLs (a total of only three). For QTLs detected by the genotypic means, the number per chromosome varied from two on chromosomes 6 and 10 to one on chromosome 1.

### QEI analysis

Multi-environment forward–backward models were used to assess the significance and the strength of the QTL effects across environments. The QEI analysis was conducted in two steps using the same set of 1345 SNP markers that were also used for the linkage mapping analysis, and yielded 28 QEI (only those showing significant interaction) for the 10 traits (Supplementary Table S4). The number of QEI varied from none for nfr to six for flw. These two traits also demonstrated the lowest and highest values of$H^{2}$, respectively.

**Fig. 4.**
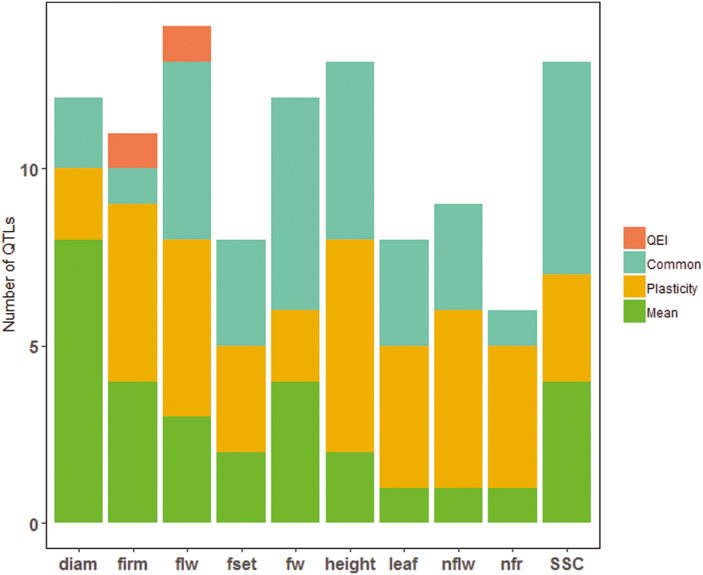
Number of QTLs identified for each trait (listed in Supplementary Table S2). The QTLs are divided into those specific to the genotypic mean (Mean), to phenotypic plasticity (Plasticity; slope, VAR, or SCv), and to QTL × environment interactions (QEI), and also QTLs that were common to at least two of these categories.

All the QEI identified in this step were compared to the plasticity and genotypic mean QTLs using the physical positions of the QTLs and their CIs. Interestingly, this comparison revealed that all the detected QEIs were also identified using either the genotypic mean or the plasticity parameters in the linkage mapping analysis, except for two QEIs located on the same region of chromosome 6 (flw6.1 and firm6.1). Among the 106 unique QTLs identified by genotypic means, phenotypic plasticity, and QEIs, a notable number were specific, with 30% and 32% for plasticity and genotypic mean, respectively (Fig. 4). Eight QTLs involving five different traits (flw1.1, fw2.1, fw2.2, fw11.2, leaf6.1, nflw11.2, SSC1.2, and SSC9.1) were identified with all the three approaches, highlighting their robustness and susceptibility to environmental variation.

### Genetic locations of the MAGIC-MET QTLs

**Fig. 5.**
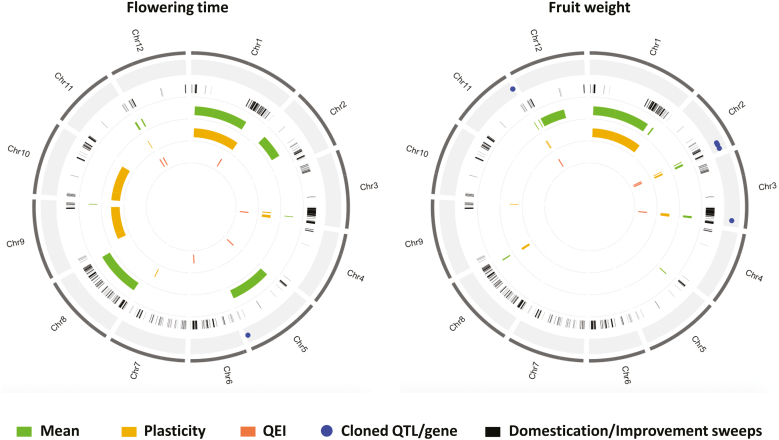
Physical positions of the MAGIC-MET QTLs for fruit weight and flowering time in the genome. The domestication/improvement sweep regions are those identified by Zhu *et al.* (2018). The QTLs are divided into those specific to the genotypic mean (Mean), to phenotypic plasticity (Plasticity; slope, VAR, or SCv), and to QTL × environment interactions (QEI). The outer circle represents the different tomato chromosomes (Chr 1–12). Next, circle (A) represents the previously cloned QTLs/genes documented in the literature, circle (B) represents the domestication/improvement sweeps (black bars), circle (C) represents the genotypic mean QTLs, circle (D) represents the plasticity QTLs, and circle (E) represents the QEIs.

The SL2.50 version of the tomato reference genome (https://solgenomics.net/) was used to compare the positions of the different QTL categories (genotypic mean, phenotypic plasticity, and QEI). A recent study has identified different regions (sweep regions) that were selected during domestication and improvement events (Zhu *et al.*, 2018), and we cross-checked these against the positions of our QTLs. Some QTLs detected in our MAGIC-MET design were located across large regions, and hence co-located with a high number of the sweep regions (Fig. 5, Supplementary Fig. S6). Considering only the QTLs with CI lower than 2 Mbp intervals and all the QEIs, a total of 61 QTLs were selected and compared with the sweep regions. The selected plasticity QTLs appeared to be in the majority of those located within the sweep regions, with only 6% being outside the domestication/improvement selective sweeps (Supplementary Fig. S7). Interestingly, the sweep region SW75 located in chromosome 3 (between 64.76–65.01 Mbp) carried a total of five QTLs (ht3.1, fset3.1, flw3.2, leaf3.1, fset3.1). All the sweep regions containing at least one of our MAGIC-MET QTLs are listed in Supplementary Table S5. Chromosome 11 was notable as containing a number of plasticity QTLs for different traits (Fig. 3). Indeed, seven different QTLs that were all identified with the plasticity parameters were located within the regions SW254 and SW255, from 53.81–55.62 Mbp on this chromosome (Supplementary Fig. S8). Among the 10 QTLs that were outside the sweep regions, one (fw5.1) was identified for mean FW and was located on chromosome 5 at 4.52 Mbp. It was mapped in a region holding other QTLs that segregated in the MAGIC population for fruit size, width, and length (Supplementary Table S6; data from Pascual *et al.*, 2015).

### Candidate genes

The CIs of the MAGIC-MET QTLs varied from 0.45–87Mbp and included a variable number of genes. We therefore focused on the QTLs presenting CI regions smaller than 2 Mbp for screening for candidate genes (CGs). Between 49 (nflw12.1) and 256 (diam4.1) genes were within the regions of the selected QTLs. Taking advantage of the parental allelic effect, the CGs were narrowed down for each QTL by contrasting the allelic effect of the eight parental lines. The selected candidates after this filtering procedure are presented in Supplementary Table S7, and represent interesting targets for further studies. For instance, flowering-time QTLs included some CGs with consistent matching with regards to their functional annotation. For example, the CI of the QTL ppflw11.1 on chromosome 11 included two CGs, namely Solyc11g070100 and Solyc11g071250, that corresponded to ‘early flowering protein’ (ELF) and ‘EMBRYO FLOWERING 1-like protein’ (EMF1), respectively. Among other potential flowering candidates, we note Solyc12g010490 (AP2-like ERF) for the QTL flw12.1, and Solyc03g114890 and Solyc03g114900 (COBRA-like proteins) for the QTL flw3.2. Apart from flowering time, the selected CGs for the QTLs diam4.1 and ppSSC1.1 included Solyc04g081870 annotated as an expansin gene, and Solyc01g006740 annotated as sucrose phosphate phosphatase gene, respectively.

We were able to identify some plasticity QTLs that showed sensitivity to the environmental conditions, notably those detected using the SCv plasticity parameter, and CGs were screened for some QTLs falling into this category. For example, the ppfw9.1 QTL CI showed susceptibility to the sum of degree-days (SDD) and carried a chaperone candidate gene (solyc09g091180) that might be involved in regulating fruit weight depending on the variation in SDD. Similarly, the QTL ppleaf11.1 was affected by the maximal temperature (Supplementary Table S4). Three CGs (Solyc11g071830, Solyc11g071930, and Solyc11g071710) belonging to the Chaperone J-domain family were retained after the filtering procedure in the region of this QTL. Interestingly, the DnaJ-like zinc-finger gene (Solyc11g071710) was among the candidates corresponding to several plasticity QTLs, including ppflw11.1, ppleaf11.1, ppnflw11.1, ppht11.1, and ppdiam11.2. This gene presented a total of 122 polymorphisms across the eight parental lines, among which 35 and 68 were in the upstream and downstream gene regions, respectively. Further investigation would be needed to determine potential pleiotropic effects of this gene.

## Discussion

### Genetic variability in the response of tomato to environmental variation

The genotype × environment interaction (G×E) represents a long-standing challenge for plant breeders, and predicted climate changes have encouraged geneticists to devote more attention to understanding its genetic basis. Tomato is a widely cultivated crop that is adapted to a variety of environmental conditions (Rothan *et al.*, 2019); however, important effects of abiotic stresses in the final productivity, fruit quality, and reproductive performance have been observed (Mitchell *et al.*, 1991; Estañ *et al.*, 2009; Albert *et al.*, 2016; Xu *et al*., 2017*b*). We quantified the level of G×E and the corresponding phenotypic plasticity in a highly recombinant tomato population in a multi-environment and multi-stress trial that included induced water-deficit, salinity, and heat stresses (Supplementary Table S1). Important genetic variability was observed for the plasticity traits related to yield, fruit quality, plant growth, and phenology (Supplementary Fig. S5), highlighting the fact that the MAGIC population represents a valuable resource for tomato breeding in dynamic stressful environments. Wild tomato species have also been characterized as an important reservoir for genes related with abiotic stress tolerance (Foolad, 2007); however, their effective use in breeding programs could be difficult due to undesirable linkage drag, notably for fruit quality. The MAGIC population characterized here is intraspecific with high diversity in terms of fruit quality components, which gives it a great advantage as a breeding resource compared to wild populations.

Several statistical models are available to explore, describe, and predict G×E in plants (Yan *et al.*, 2007; Malosetti *et al.*, 2013). Factorial regression models are among the most attractive as they enable the description of observed G×E in relation to relevant environmental information. We used a factorial regression model with different environmental covariates that were readily accessible from year to year, which allowed us to predict a variable proportion of the observed G×E (Supplementary Fig. S4). In addition, each MAGIC line was characterized for its sensitivity to the climatic conditions during growth, thus opening avenues to efficiently select the most interesting genotypes for further evaluation in breeding programs targeting stressful environments.

Interestingly, we found significant correlations between the genotypic sensitivities to the different environmental covariates and the slopes of the Finlay–Wilkinson regression model (Supplementary Fig. S9). This emphasizes the adequacy of the selected environmental covariates to explain the differences observed in the average performance of the genotypes across environments. In contrast, slope and VAR showed correlations that were less significant, although they were both correlated to the mean phenotypes in the same direction, except for SSC (Fig. 2). This may have been induced by distinct genetic regulation of these two plasticity parameters, which reflect different types of agronomic stability (Lin *et al.*, 1986). Indeed, we identified 7 and 14 plasticity QTLs that were specific to VAR and slope, respectively (Supplementary Table S4). The correlation pattern of the different plasticity parameters thus suggests a complex regulation of plasticity, which is also seemingly trait specific.

Significant correlation at the phenotypic level might result from the action of pleiotropic genes. The correlations between genotypic means and plasticity that were significant for almost every trait to a variable degree are shown in Fig. 2. These correlations were reflected at the genetic level by 22 QTLs that overlapped between the genotypic mean and the plasticity parameters, representing ~21% of all the identified QTLs. However, a high proportion of the QTLs were specific either to the genotypic means or the plasticity parameters (Supplementary Fig. S10), hence suggesting the action of both common and distinct genetic loci in the control of mean phenotype and plasticity variation in tomato.

### Genomic location of the MAGIC-MET QTLs

The availability of substantial genomic information in tomato has enabled the identification of different genomic regions that have undergone selective sweeps and that have been strongly selected for during the process of crop domestication and improvement (Lin *et al.*, 2014; Zhu *et al.*, 2018). When projected onto the physical positions of the tomato reference genome (SL2.50 version), most of the plasticity QTLs we identified were located within the sweep regions defined by Zhu *et al.* (2018). This therefore suggests that plasticity might have been selected together with other interesting agronomic traits during tomato domestication and improvement. This is corroborated, for example, by the positive correlation between the slope from the Finlay–Wilkinson regression model and the variation in mean fruit weight (Fig. 2). Indeed, genotypes with a greater slope for fruit weight were characterized by good adaptability in high-quality environments, and this is probably attributable to selection. Co-selection of allelic variants that lead to improved performance in optimal conditions together with alleles for plasticity provides a realistic assumption that would explain the significant correlation that we observed between the genotypic means and plasticity (Fig. 2). *GhD7* in rice has been reported to be a key high-yield gene that is simultaneously involved in both the regulation of plasticity of panicle and tiller branching and in the abiotic stress response (Herath, 2019). This provides an example of a gene carrying different allelic variants that affects both plasticity and the mean phenotype. Further investigations are needed to assess how domestication and breeding have affected plasticity in tomato and other crop species.

An important genomic region involved in the genetic regulation of plasticity for six different traits was identified in chromosome 11 (Supplementary Fig. S8). This region is obviously a regulatory hub that carries interesting plasticity genes. It remains to determine whether the co-localization of the different plasticity QTLs in this region is due to the action of a pleiotropic gene or of different linked genes. Nevertheless, this chromosome 11 region is an interesting target for breeding as well as for understanding the functional mechanisms of plasticity genes.

### Allelic-sensitivity versus gene-regulatory models

We identified 63 plasticity QTLs (Supplementary Table S4), among which 22 (35%) were also identified when using the genotypic means, and 41 (65%) were specific to plasticity. Via *et al.* (1995) proposed two models among the mechanisms involved in the genetic control of phenotypic plasticity, namely the allelic-sensitivity and gene-regulatory models. These models are distinguishable through QTL analysis (Ungerer *et al.*, 2003), with the expectation that the allelic-sensitivity model will lead to co-localization of genotypic means and plasticity QTLs, whereas a distinct location of QTLs that affect the genotypic mean and plasticity will probably correspond to the gene-regulatory model (Kusmec *et al.*, 2017). In relation to our results, tomato plasticity appeared to fall within both models, although the gene-regulatory model was predominant, with 65% of the QTLs for plasticity not co-localizing with the QTLs for genotypic means for the same trait (Supplementary Table S4). Kusmec *et al.* (2017) found similar results in maize using a larger number of environments and traits, and identified an even higher rate of distinct locations of QTLs for plasticity and genotypic means. Studying plasticity as a trait *per se* is therefore of major interest since breeding in both directions (considering the mean phenotype and its plasticity) is achievable. Through transcriptomic analyses, Albert *et al.* (2018) observed that the genotype × water deficit interaction in tomato was mostly associated with *trans-*acting genes and could be assimilated within the gene-regulatory model, in agreement with our results.

**Fig. 6.**
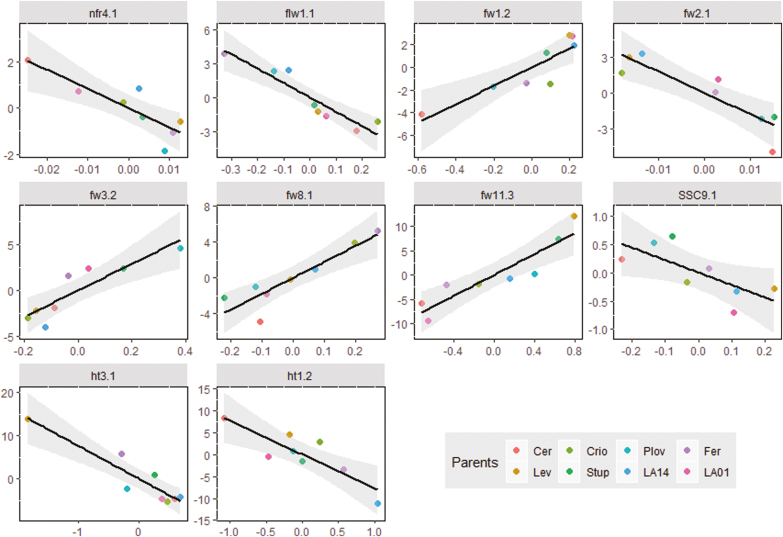
Correlations between the estimated allelic effects for consistent QTLs between the genotypic means and plasticity phenotypes for each trait (listed in Supplementary Table S2) for the eight parental lines of the MAGIC population.

Although the distinct location of QTLs detected for plasticity and genotypic mean could be confidently assigned to the interaction of genes, their co-localization is not necessarily a case of allelic-sensitivity regulation, especially if the QTL is in a large region. Indeed, the allelic-sensitivity model assumes that a constitutive gene is directly sensitive to the environment that regulates its expression across different environmental conditions, hence inducing phenotypic plasticity. This is a very strong hypothesis regarding QTLs since the overlapping region between genotypic means and plasticity could carry different causal variants in strong linkage disequilibrium that affect either the mean phenotype or plasticity. Thus, co-locating mean and plasticity QTLs should be not automatically imputed to the allelic-sensitivity model. We found a total of 22 constitutive QTLs between genotypic means and plasticity for all 10 measured traits (Supplementary Table S4). Considering the estimated QTL effects, the variation patterns of the eight parental allelic classes were compared between the genotypic mean and phenotypic plasticity QTL of the same trait. Only 10 QTLs showed consistent allelic effects (Spearman correlation significant at *P*<0.05), thus strengthening the hypothesis of the allelic-sensitivity model for these QTLs (Fig. 6). Further studies should help to identify and validate the candidate plasticity genes and to clarify their functional mechanisms.

### Complementary methods to identify environment-responsive QTLs

Different approaches have been proposed to dissect G×E into its genetic components (Malosetti *et al.*, 2013; El-Soda *et al.*, 2014). We used mixed linear models with a random genetic effect accounting for the correlation structure of the MAGIC-MET design (eqns 5, 6) to identify QTL × environment interactions (QEIs). Extending the use of mixed linear models to MAGIC populations in the framework of MET analysis has very rarely been applied in crops. To our knowledge, only Verbyla *et al.* (2014) have used such an approach, and they identified QEIs for flowering time in wheat. Our model was adequate to account for the complex mating design of the MAGIC population by using the haplotype probabilities. Indeed, it allowed estimation of the QTL effect for each parental allelic class and for each environment at every SNP marker. Overall, 28 QEI were detected that showed significant marker × environment interactions for 10 traits (Supplementary Table S4).

Methods using plasticity as a trait *per se* are also attractive for identifying environmentally sensitive QTLs. This strategy has been applied in maize, sunflower, barley, and soybean to detect the loci governing G×E (Lacaze *et al.*, 2009; Gage *et al.*, 2017; Kusmec *et al.*, 2017; Mangin *et al.*, 2017; Xavier *et al.*, 2018). Using different parameters, we identified a total of 63 plasticity QTLs and only 24% were also identified with the QEI models (Supplementary Fig. S10). Thus, using both plasticity and mixed linear models are complementary approaches to study the genetic components of G×E.

### Candidate genes

Multi-parental populations are powerful tools for QTL mapping studies (Kover *et al.*, 2009; Huang *et al.*, 2012) and in addition are interesting for fine mapping and screening of candidate genes. For example, Barrero *et al.* (2015) considered the variation of the QTL effect estimated for the different parental lines combined with transcriptomic analyses to efficiently identify candidate genes in wheat. Similarly, Septiani *et al.* (2019) narrowed down the candidate genes for *Fusarium* resistance in a maize MAGIC population using the allelic effects of the MAGIC parents.

Our study identified a number of potential candidate genes (CGs), affecting both the genotypic means and plasticity variation (Supplementary Table S7). These CGs were selected based on the parental allelic effects and represent valuable targets for future studies attempting to characterize the molecular mechanisms underlying plasticity in tomato. Specifically, relevant CGs were identified for plasticity of flowering time, including Solyc11g071250 that corresponds to an ‘EMBRYO FLOWERING 1-like’ (EMF1) protein. The involvement of EMF1 in flowering time in Arabidopsis has been reported by Aubert *et al.* (2001), who highlighted an indirect effect on flowering time and inflorescence architecture. More recently, Luo *et al.* (2018) described the role of EMF1 in interactions with CONSTANS proteins in a complex pathway to regulate the expression of flowering-time genes in Arabidopsis. Solyc11g070100, which is annotated as a ‘Early flowering protein’ (ELF) gene, is also an interesting candidate for flowering-time regulation. It has been reported that consistent expression of *ELF3* across a number of species can extend the rapid transition to flowering (Huang *et al.*, 2017), leading the authors to conclude that its loss of function would therefore be expected to trigger early flowering. Interestingly, we observed that Solyc11g070100 was affected by 69 SNPs and 14 INDEL polymorphisms, among which only one SNP showed polymorphism variation in line with the estimated allelic effect for the eight parental lines at this QTL. This SNP was localized at position 54 632 225 bp in chromosome 11, upstream of Solyc11g070100. The parent LA1420 carried the reference allele at this SNP while the remaining parents held the alternative allele. Considering the estimated allelic effects at this QTL, we can assume that the LA1420 allele variant might induce an early flowering phenotype in comparison to the other parents.

## Conclusions

We aimed to dissect the genetic architecture of the responses of tomato to different environments by imposing a variety of abiotic stresses at different geographic locations on a multi-parental advanced generation intercross (MAGIC) population. The population demonstrated a large genetic variability in response to the stresses, which was reflected in the identification of 63 QTLs for plasticity. This was achieved through the use of different plasticity parameters, thus highlighting the importance of quantifying plasticity in order to be able to decipher its genetic basis. The majority of the plasticity QTLs (65%) were located in different regions to the QTLs that were detected for the mean phenotypes, suggesting that there is to some extent specific genetic control of mean trait variation and plasticity. Using plasticity as a trait *per se* in mapping analysis turned out to be a good method for identifying genetic regions underlying genotype × environment interactions. Almost all the QTL × environment interactions (QEIs) were identified for at least one of the plasticity parameters as well. Overall, our study highlights the MAGIC population as a powerful resource for tomato breeding under abiotic stress conditions, as well as for understanding the genetic mechanisms that underlie the regulation of the response to tomato to environmental variation.

## Supplementary data

Supplementary data are available at *JXB* online.

Fig. S1. Selection of the seven environmental covariates for the factorial regression model.

Fig. S2. Boxplot of the distribution of the traits across the different environments.

Fig. S3. Heritability of traits in the MAGIC-MET design.

Fig. S4. Proportions of the sum-of-squares attributed to the different factors in the factorial regression model.

Fig. S5. Histograms of the distribution of the means and all plasticity parameters for each trait

Fig. S6. Physical positions of the MAGIC-MET QTLs on the chromosomes.

Fig. S7. Number of the MAGIC-MET QTLs identified within and outside domesticated/improved selective sweep regions.

Fig. S8. Zoom plot of the region from 53–57 Mbp on chromosome 11.

Fig. S9. Correlations between the genotypic sensitivities to environmental covariates from the factorial regression model and the slopes from the Finlay–Wilkinson regression model.

Fig. S10. Venn diagram of the number of QTLs specifically or commonly detected using the genotypic means, phenotypic plasticity, or the QEI models.

Table S1. Description of the MAGIC-MET design with the 12 environments and their respective names.

Table S2. Description of the phenotypic traits evaluated in the MAGIC-MET design.

Table S3. Estimates of the variance components from eqn (2).

Table S4. Results of QTL and QEI analyses in the MAGIC-MET design.

Table S5. Genetic location of MAGIC-MET QTLs that overlap with domestication/improvement selective sweep regions.

Table S6. QTLs identified for fruit size, width, and length in the MAGIC population.

Table S7. Selected candidate genes for all the genotypic means and plasticity QTLs located within the 2-Mbp CI region.

eraa265_suppl_Supplementary_Figures-S1-S10

eraa265_suppl_Supplementary_Tables-S1-S7
