# Supplementary material for: Genetic basis of phenotypic plasticity and genotype × environment interactions in a multi-parental tomato population
Source: J Exp Bot. 2020 May 31;71(18):5365–76. doi: 10.1093/jxb/eraa265 (PMC7501811; doi:10.1093/jxb/eraa265)

Supplemental Figure 1: Selection of 7 environmental covariates for the factorial regression model

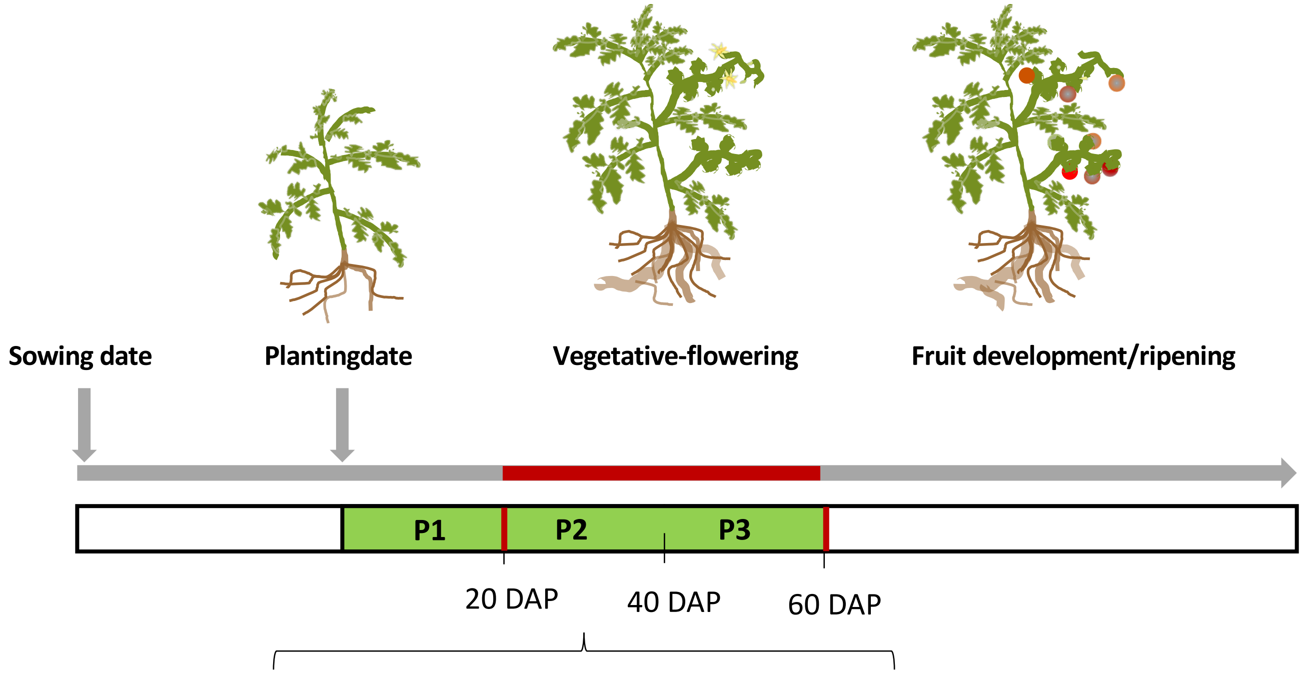

- Average daily temperatures (mean – minimal – maximal)
- Daily thermal amplitude (T° max – T° min)
- Relative humidity (24 hours)
- Vapour pressure deficit *(Abtew & Melesse 2013)*
- Sum of degree day *(SDD= [(Tmin+Tmax)/2] - Tbase10)*

⇨ 7 environmental covariates

DAP = Days after plantation

Period covering the flowering time (under the 4th truss) of the MAGIC population

Supplemental Figure 2: Boxplot distribution of the traits across environments. The colors of the boxplot are according to the groups defined by clustering of the environments

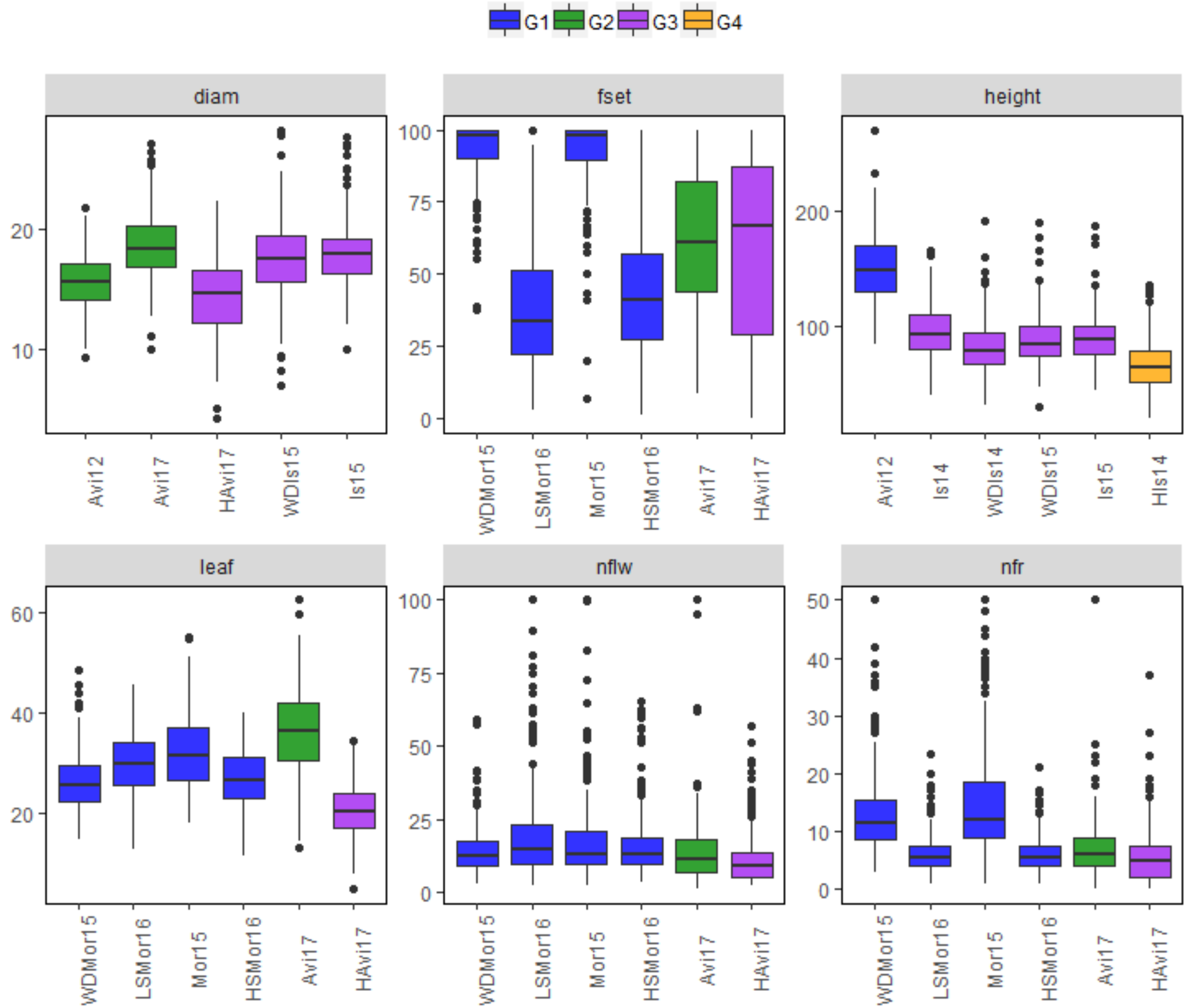

Supplemental Figure 2: Boxplot distribution of the traits across environments. The colors of the boxplot are according to the groups defined by clustering of the environments

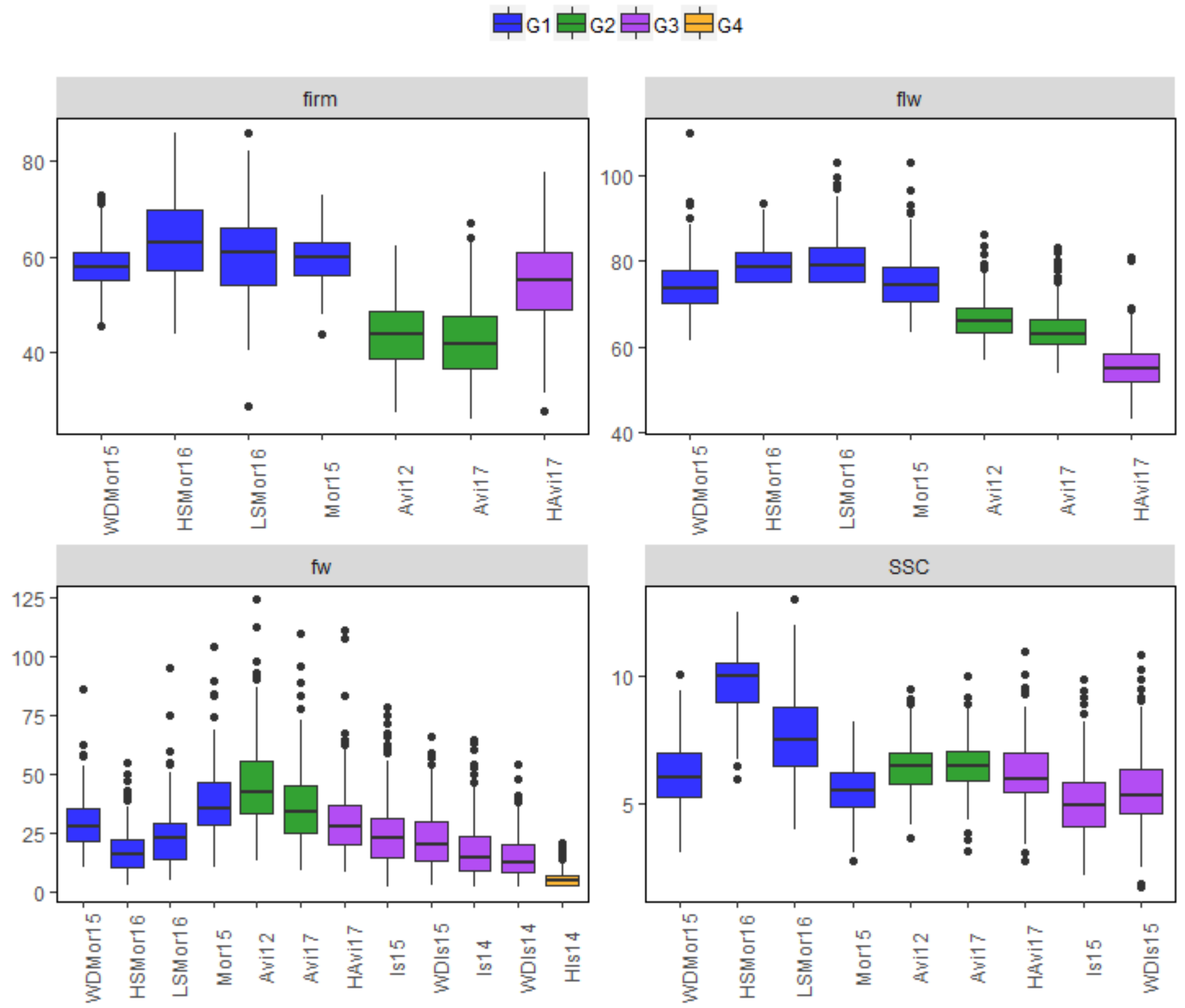

Supplemental Figure 3: Heritability in the MAGIC-MET design. For each trait, heritability was computed at every environment and plotted with heritability of the full design $\boldsymbol{H^2}$ (in green)

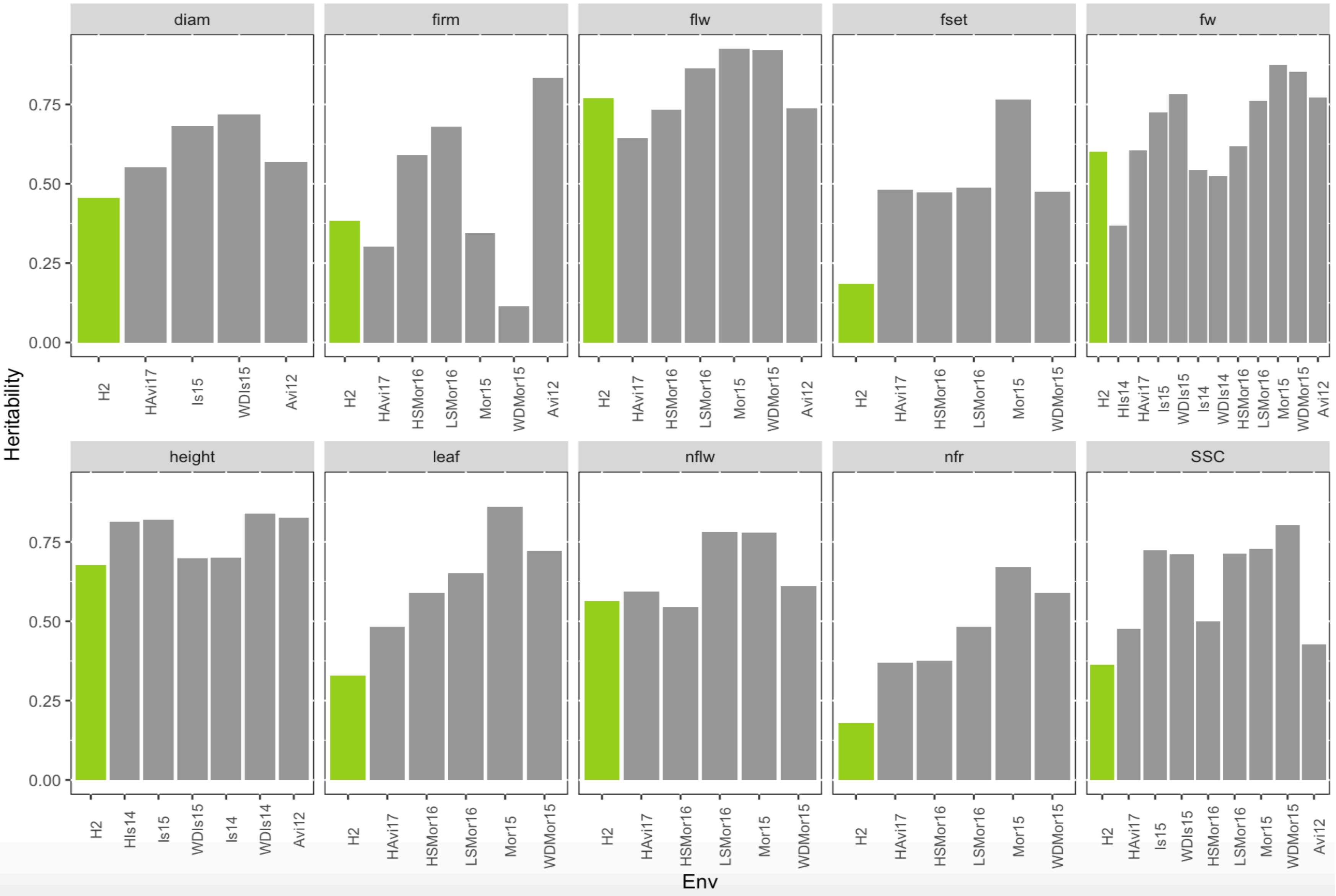

Supplemental Figure 4: Proportion of the sum of square attributed to the different factors in the factorial regression model. For each trait, the orange and green stacked bars represent the proportion of the SSq explained by the Genotype and Environment factors in a modified version of model (4) where all environmental covariates have been considered. The remaining colors represent the part of the GxE that could be explained by the different environmental covariates. Stars within the different bars highlighted the significant covariates ($\alpha < 0.05$). The missing bars for some traits denote the covariates that accounted for no variance.

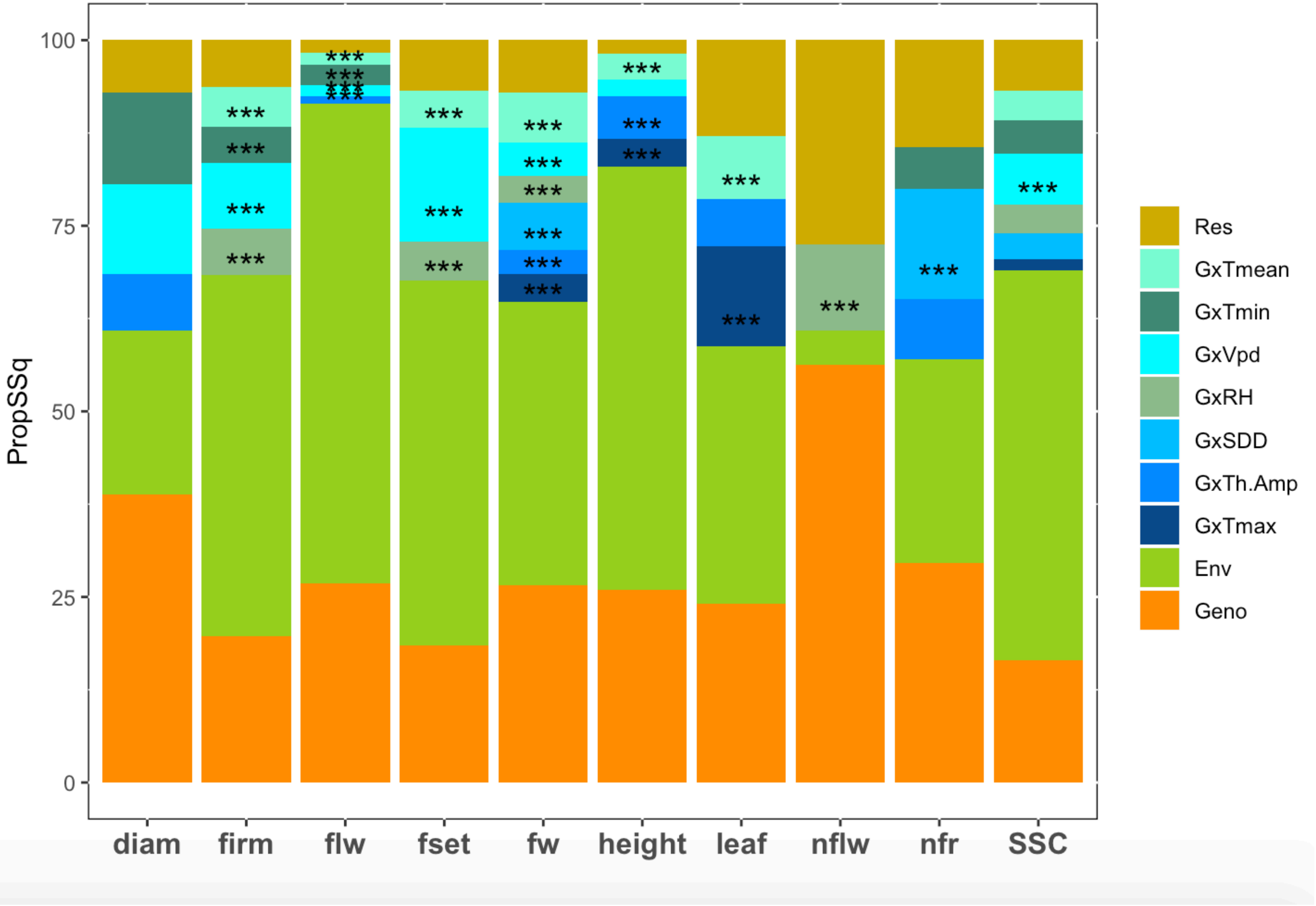

Supplemental Figure 5: Histogram distribution of mean and all plasticity parameters for each trait

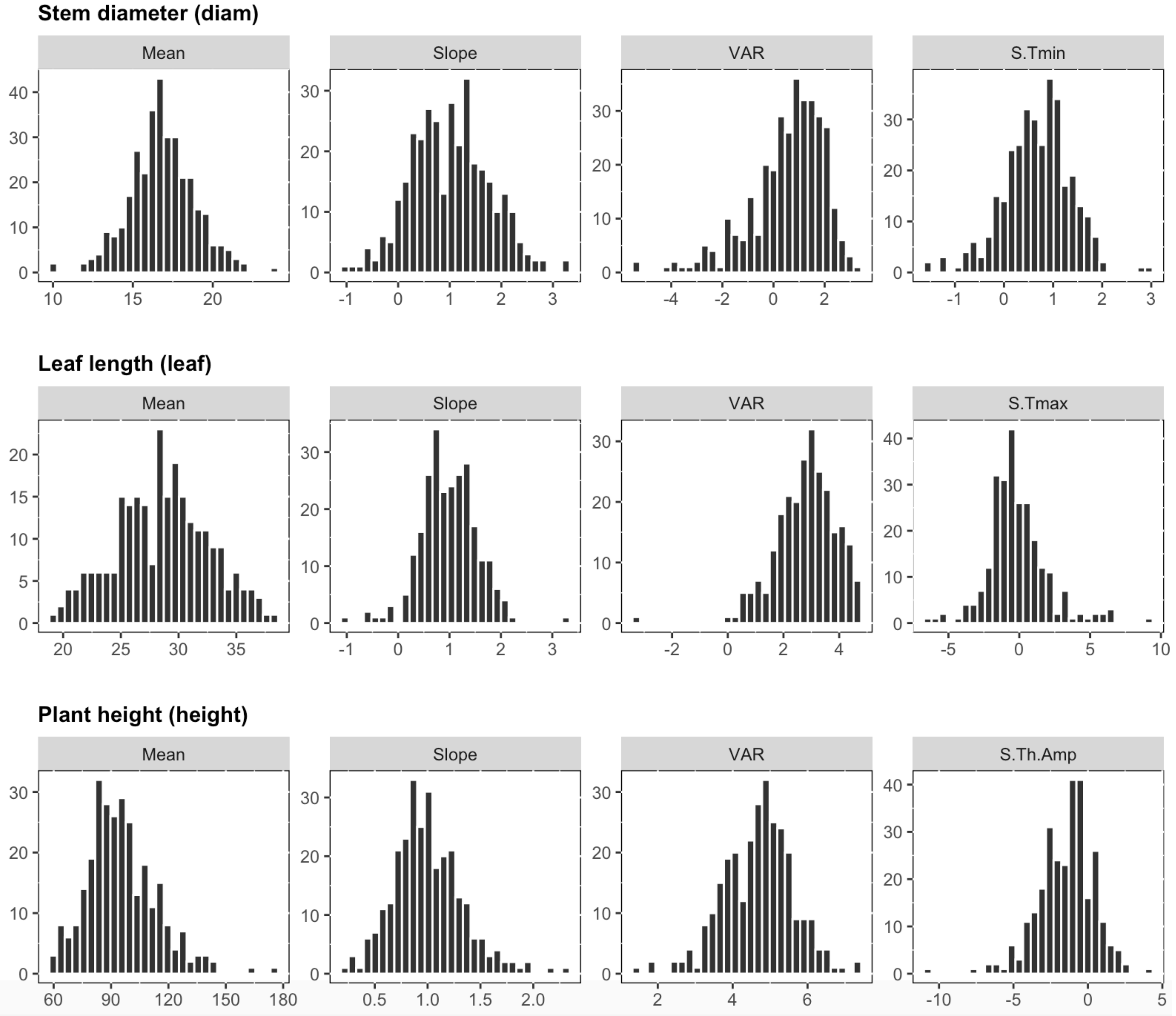

Supplemental Figure 5: Histogram distribution of mean and all plasticity parameters for each trait

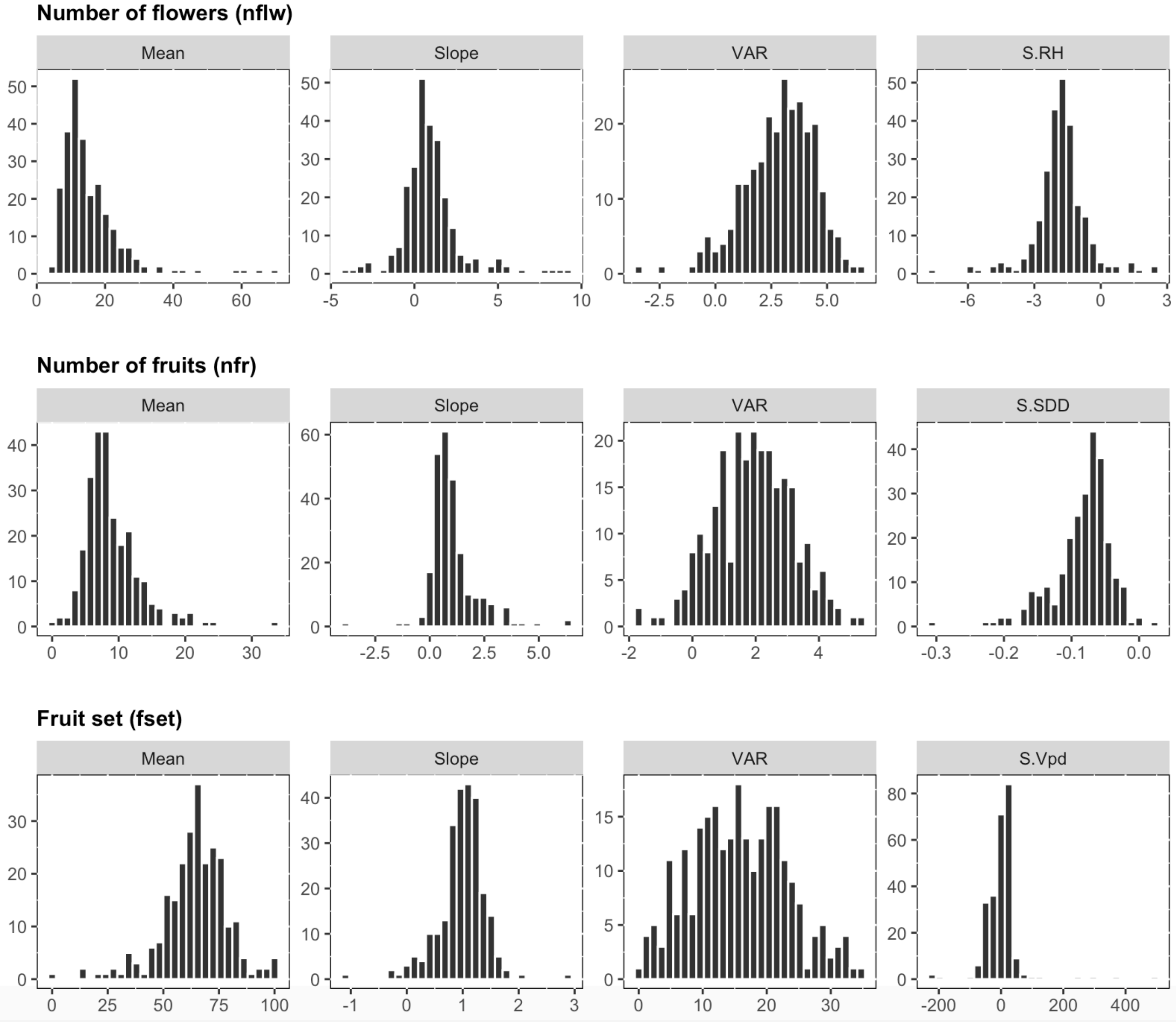

Supplemental Figure 5: Histogram distribution of mean and all plasticity parameters for each trait

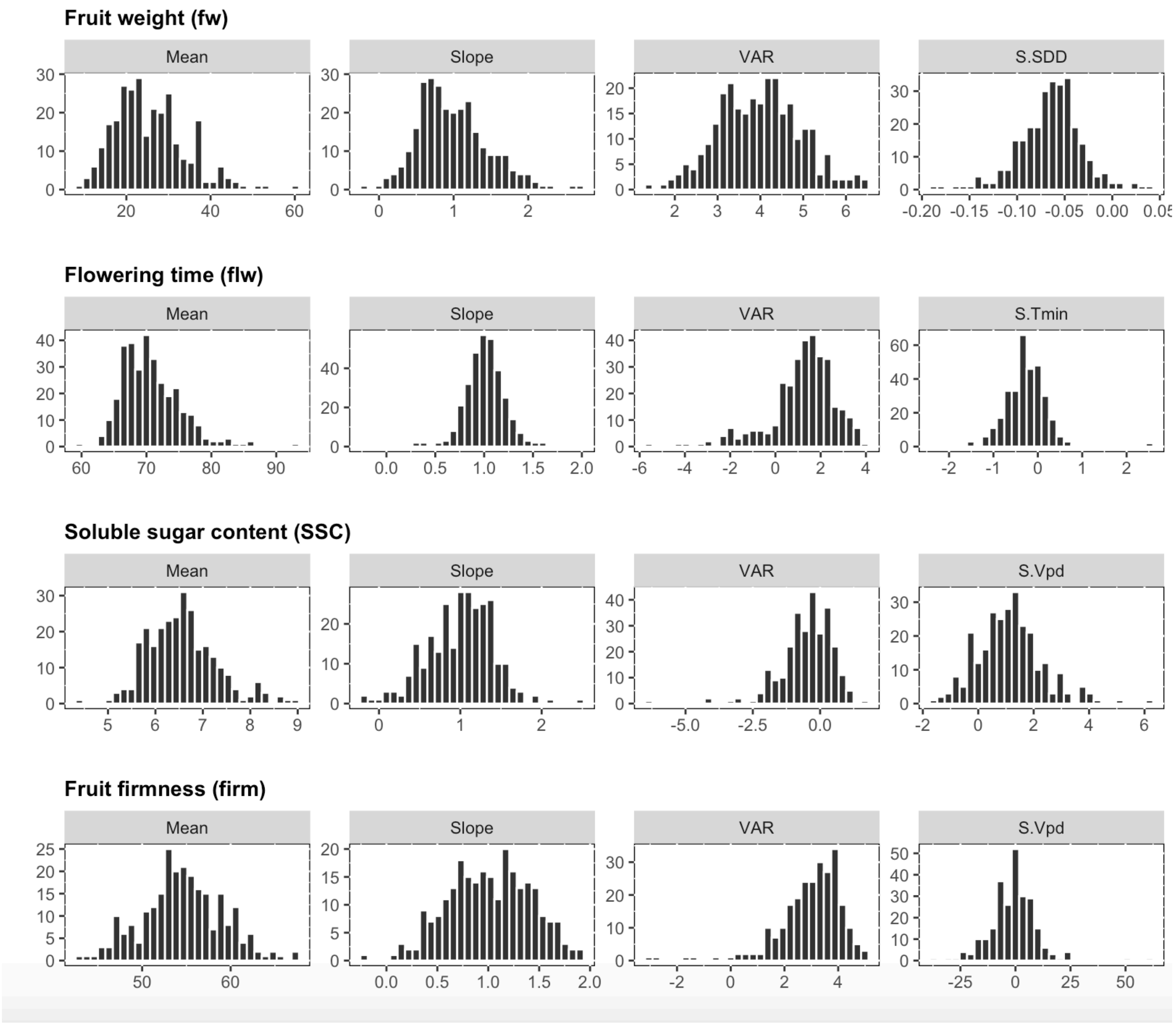

Supplemental Figure 6: Physical positions of the MAGIC-MET QTLs for diam, leaf, height, fset, nflw, nfr, firm and SSC. The outer circle with gray font represents the known and cloned QTL/gene for each trait. The following circle with black bars represents the different domestication/improvement sweep regions identified in (Zhu et al. 2018). The other circles plot the CI of QTLs identified on mean, plasticity or with QEI analysis

Stem diameter (diam)

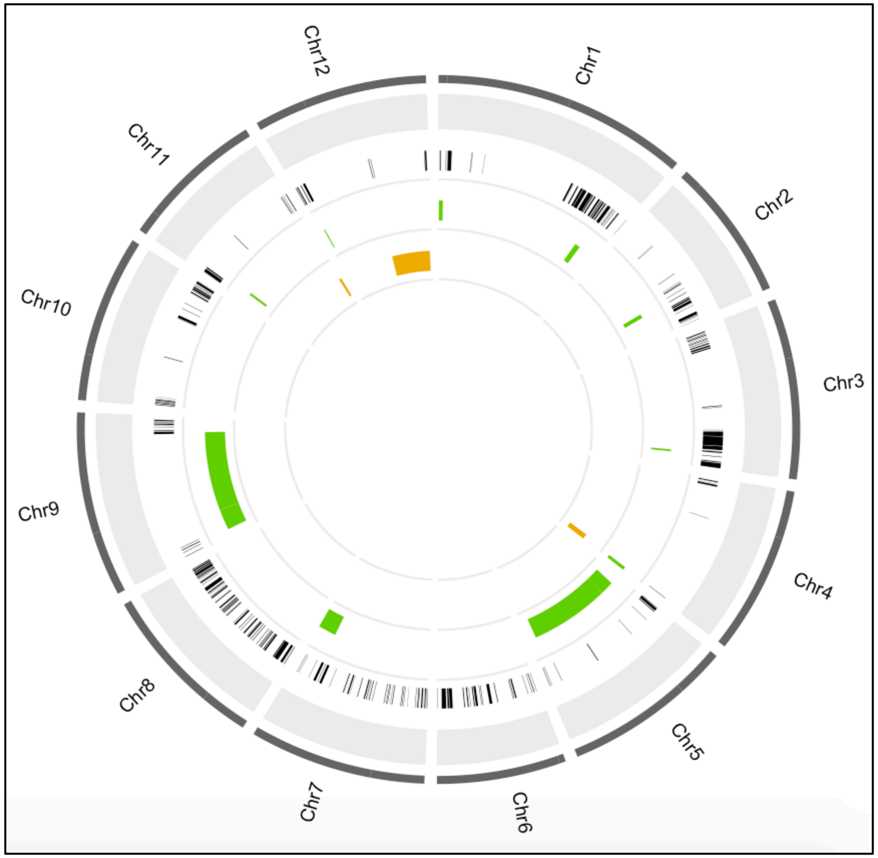

Leaf length (leaf)

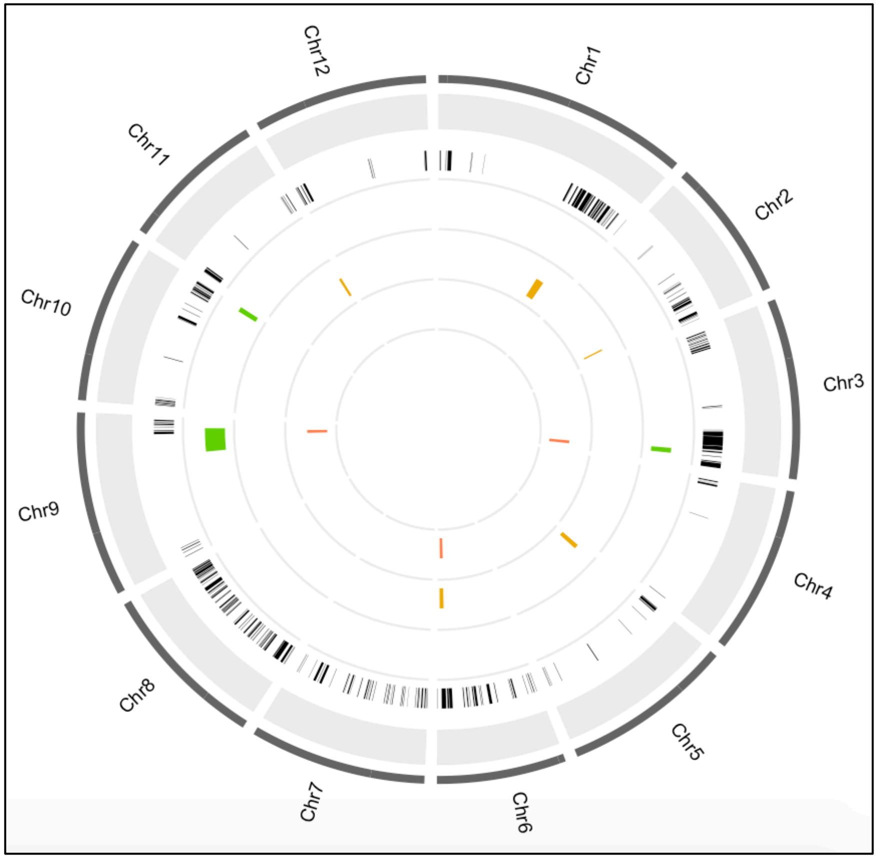

Mean **Plasticity** **QEI** **Cloned QTL/gene** **Domestication/Improvement sweeps**

Supplemental Figure 6:

Plant height (height)

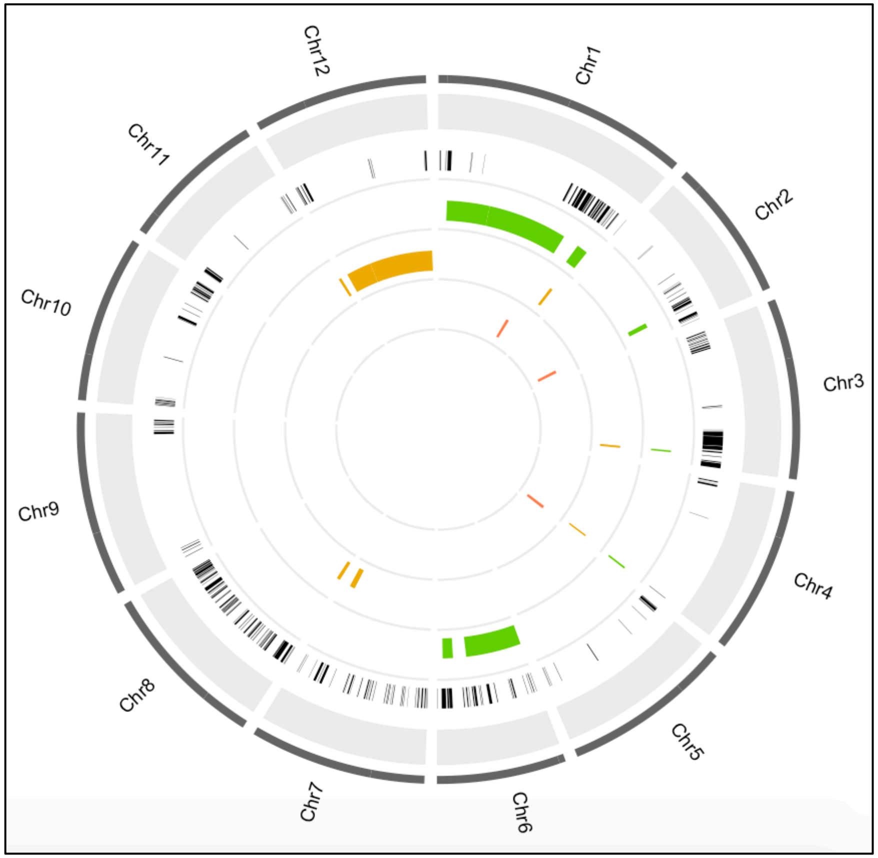

Fruit set (fset)

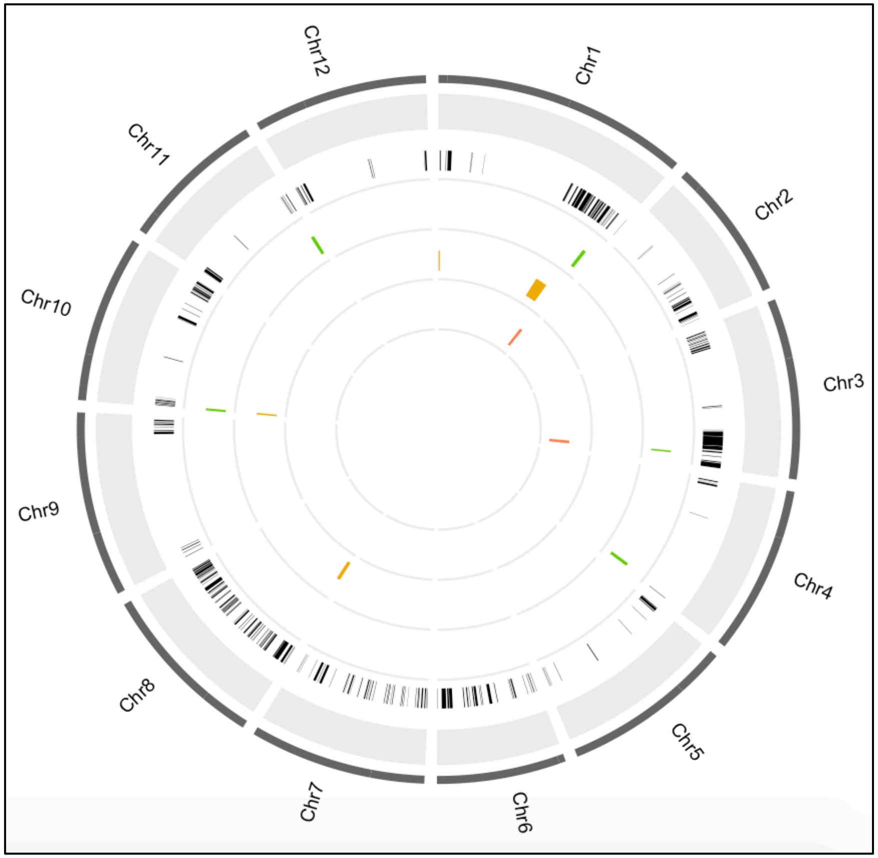

Mean Plasticity QEI Cloned QTL/gene Domestication/Improvement sweeps

Supplemental Figure 6:

Number of flowers (nflw)

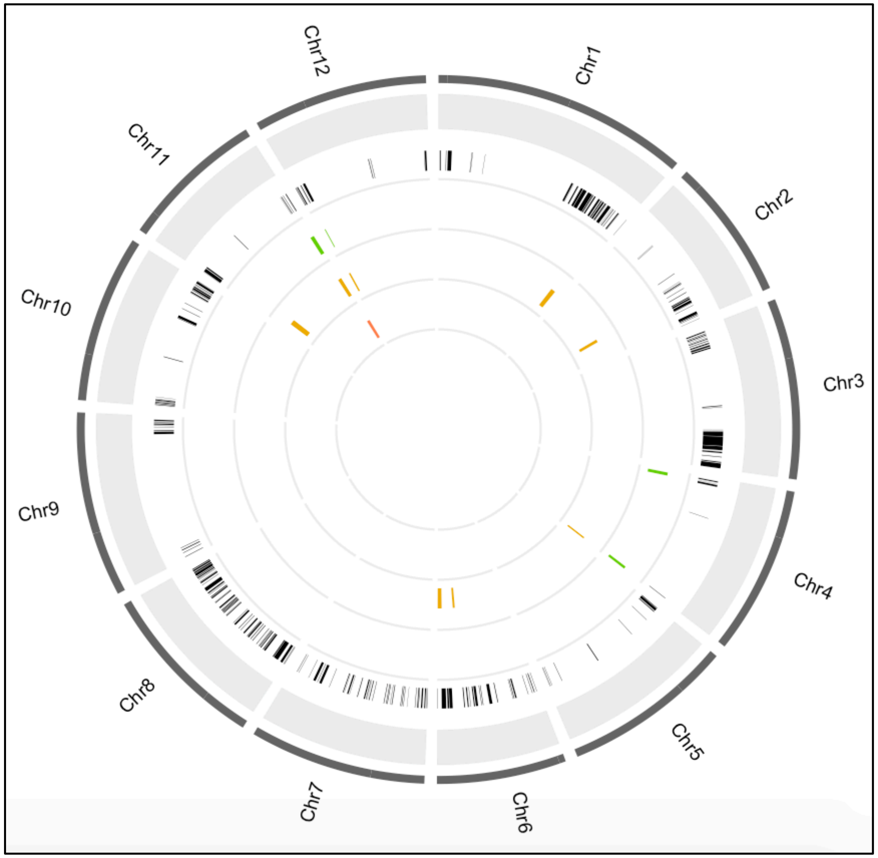

Number of fruits (nfr)

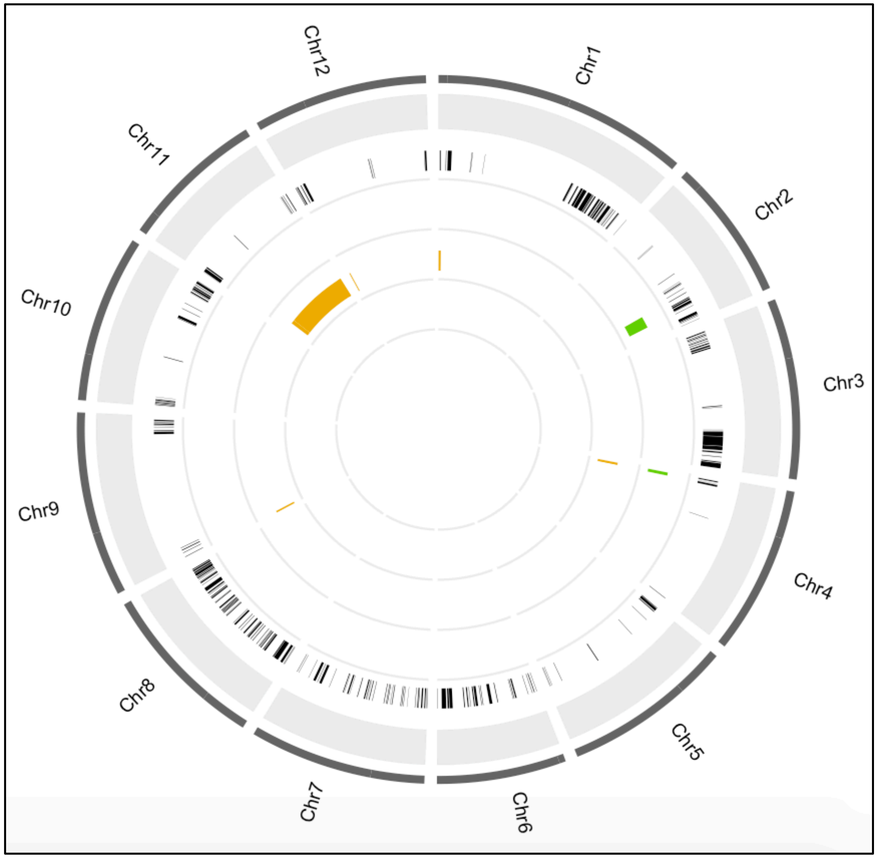

Mean **Plasticity** **QEI** **Cloned QTL/gene** **Domestication/Improvement sweeps**

Supplemental Figure 6:

Fruit firmness (firm)

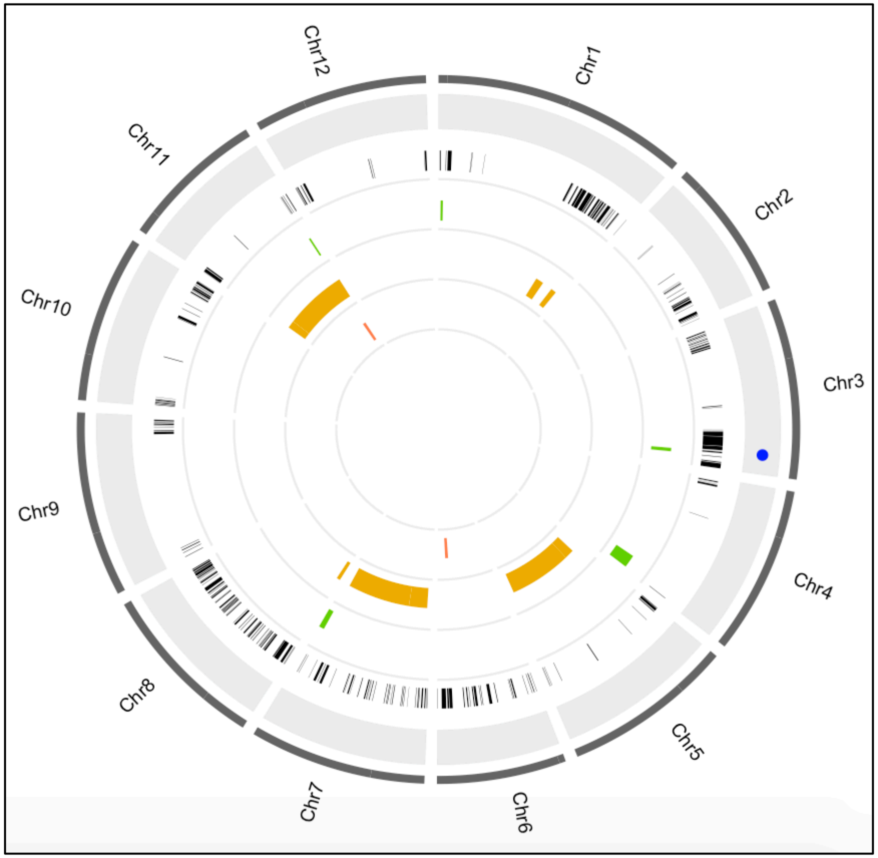

Soluble solid content (SSC)

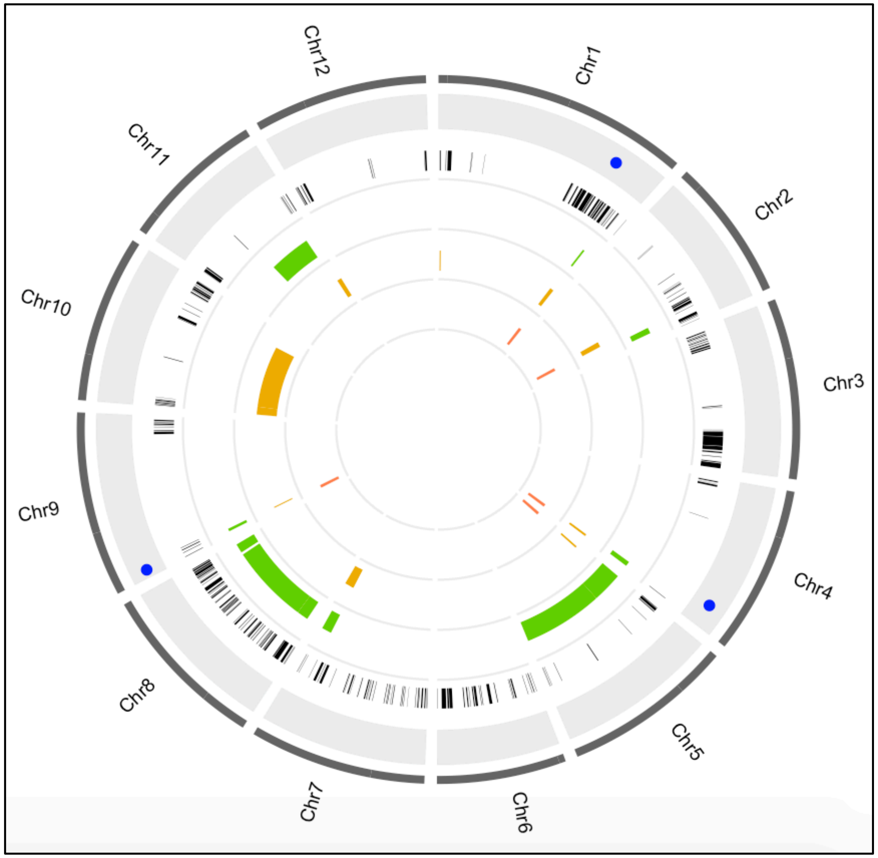

Mean **Plasticity** **QEI** **Cloned QTL/gene** **Domestication/Improvement sweeps**

Supplemental Figure 7: Number of the MAGIC-MET QTLs identified within or outside the domesticated/improved regions. Only the MAGIC-MET QTLs within short CI (lower than 2Mbp) were considered. The response specific category included QEI and plasticity specific QTLs; the common category correspond to QTLs that were commonly identified on mean, plasticity and QEI or at least two of them

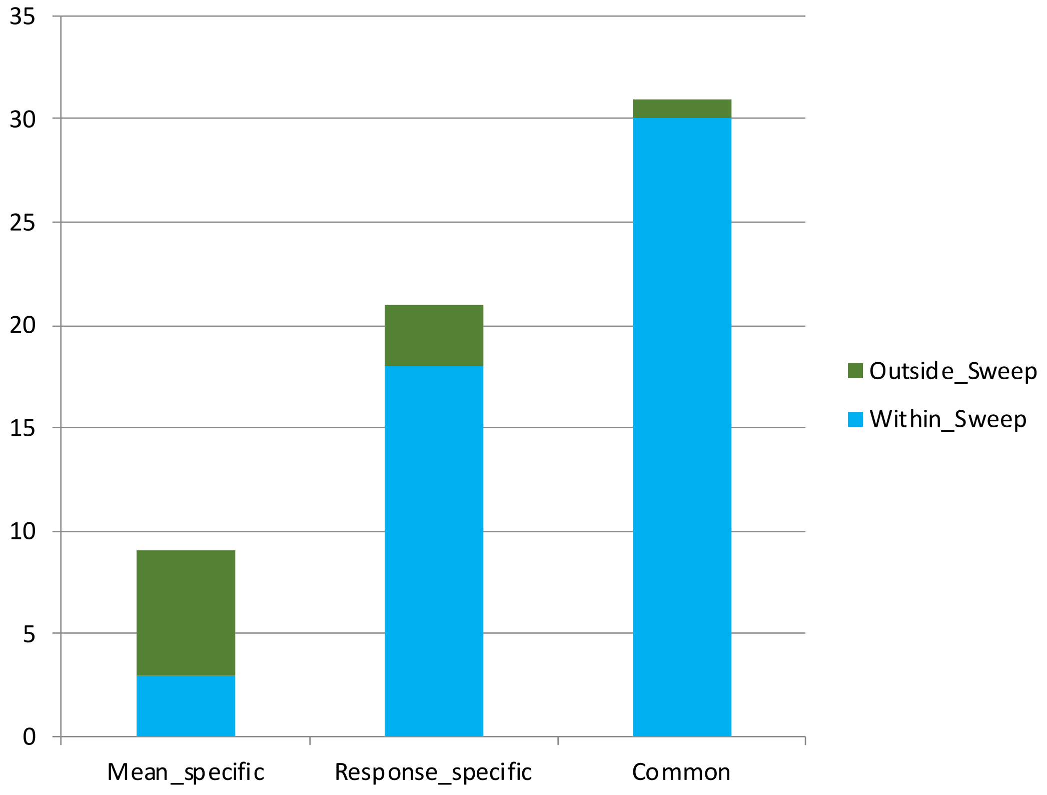

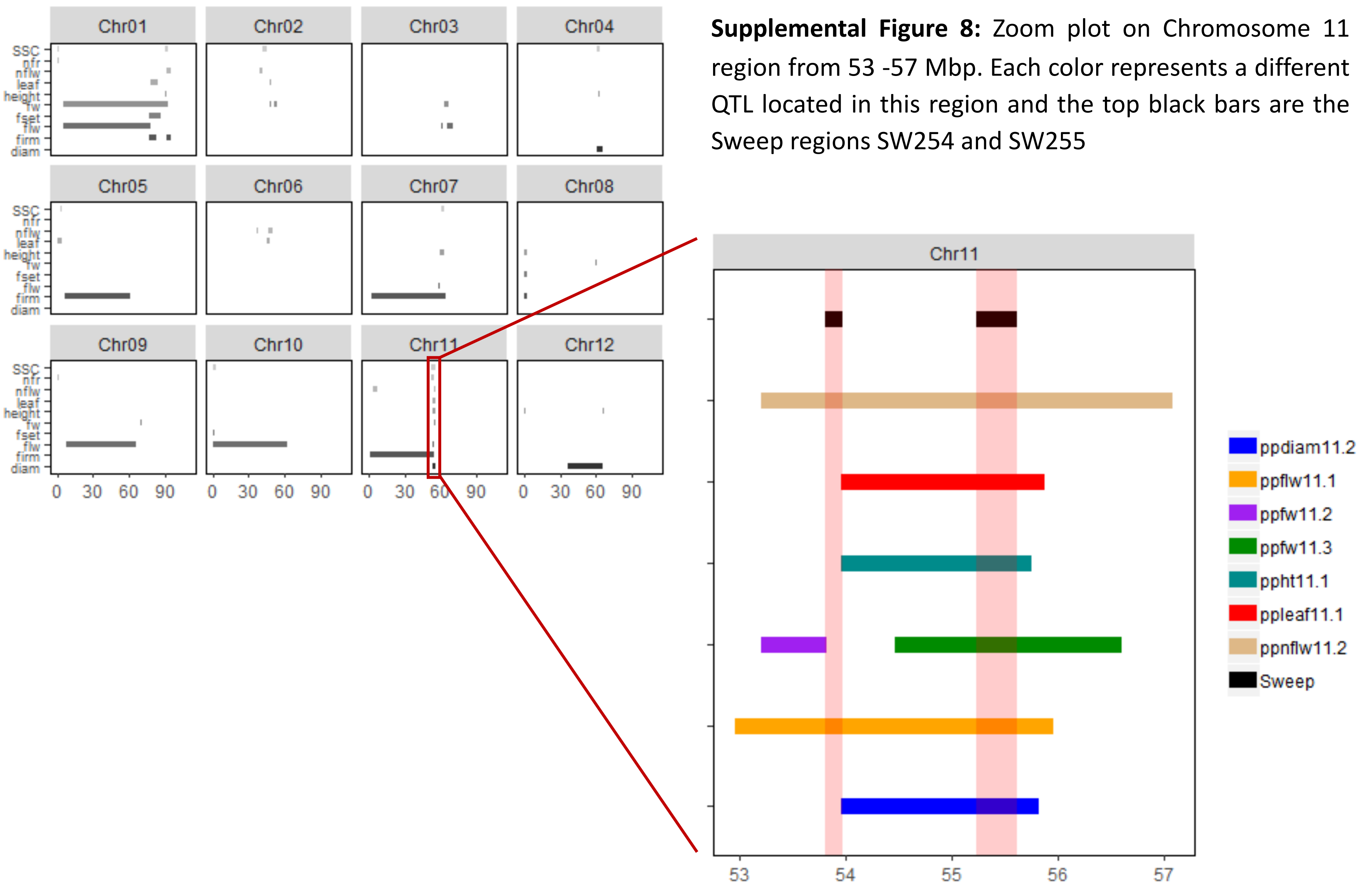

Supplemental Figure 8: Zoom plot on Chromosome 11 region from 53 -57 Mbp. Each color represents a different QTL located in this region and the top black bars are the Sweep regions SW254 and SW255

Supplemental Figure 9: Correlation between the genotypic sensitivities to environmental covariates from the factorial regression model and slopes from the Finlay-Wilkinson regression model. The negative correlations between ”slopes” and “Scv” are an artifact of the analysis due to differences in the order of environments between average performance and the chosen environmental covariates.

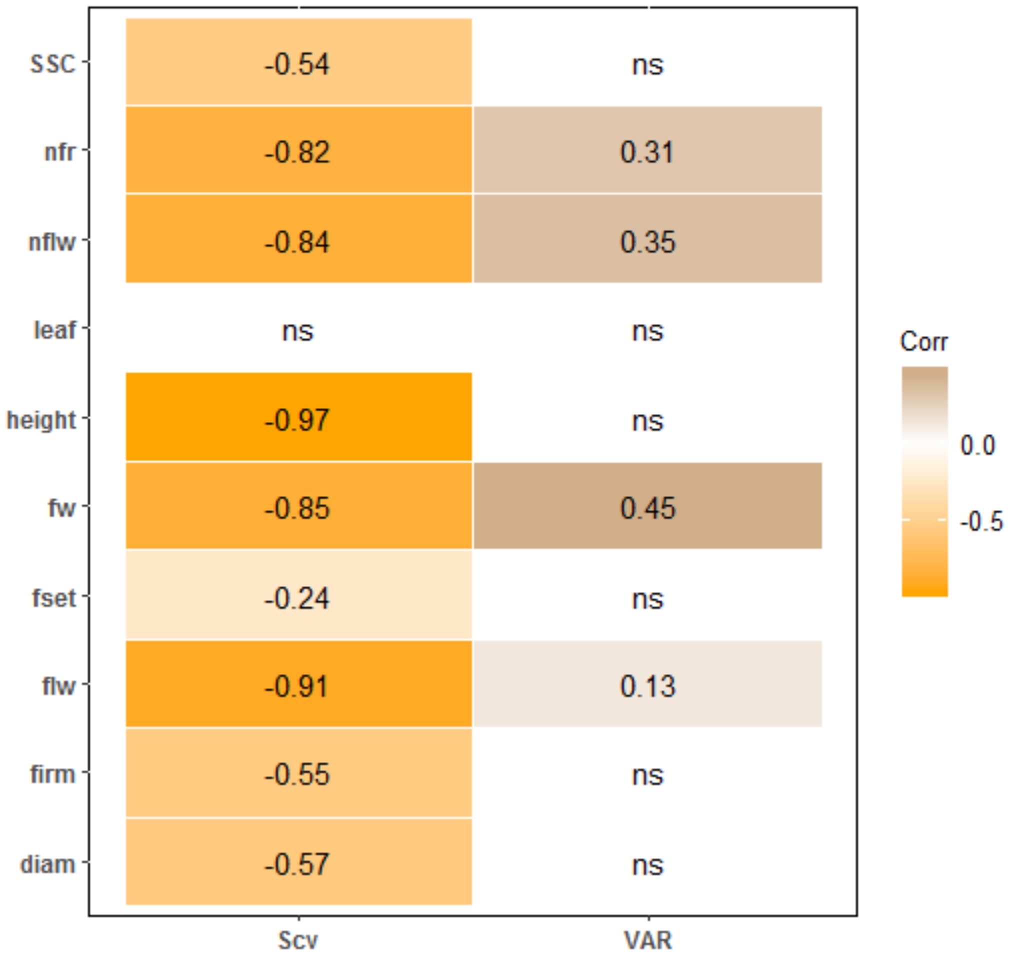

Supplemental Figure 10: Venn diagram of the number of QTL specific or commonly detected with mean, PP or using the QEI models.

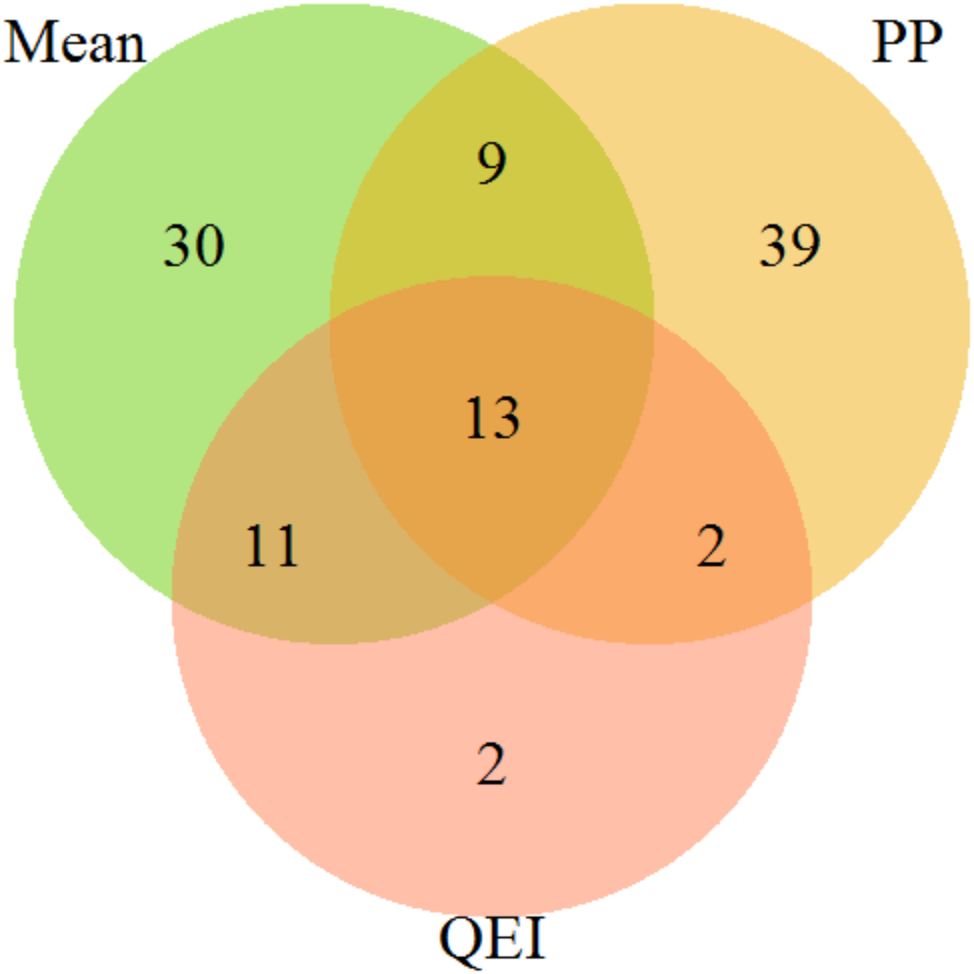

Supplement: eraa265_suppl_Supplementary_Figures-S1-S10 [file eraa265_suppl_supplementary_figures-s1-s10.pdf]
